# Formation of VEGF isoform-specific spatial distributions governing angiogenesis: computational analysis

**DOI:** 10.1186/1752-0509-5-59

**Published:** 2011-05-02

**Authors:** Prakash Vempati, Aleksander S Popel, Feilim Mac Gabhann

**Affiliations:** 1 Department of Biomedical Engineering, Johns Hopkins University School of Medicine, Baltimore, Maryland, 21205 USA; 2 Institute for Computational Medicine and Department of Biomedical Engineering, Johns Hopkins University, Baltimore, Maryland, 21218 USA

## Abstract

**Background:**

The spatial distribution of vascular endothelial growth factor A (VEGF) is an important mediator of vascular patterning. Previous experimental studies in the mouse hindbrain and retina have suggested that VEGF alternative splicing, which controls the ability of VEGF to bind to heparan sulfate proteoglycans (HSPGs) in the extracellular matrix (ECM), plays a key role in controlling VEGF diffusion and gradients in tissues. Conversely, proteolysis notably by matrix metalloproteinases (MMPs), plays a critical role in pathological situations by releasing matrix-sequestered VEGF and modulating angiogenesis. However, computational models have predicted that HSPG binding alone does not affect VEGF localization or gradients at steady state.

**Results:**

Using a 3D molecular-detailed reaction-diffusion model of VEGF ligand-receptor kinetics and transport, we test alternate models of VEGF transport in the extracellular environment surrounding an endothelial sprout. We show that differences in localization between VEGF isoforms, as observed experimentally in the mouse hindbrain, as well as the ability of proteases to redistribute VEGF in pathological situations, are consistent with a model where VEGF is endogenously cleared or degraded in an isoform-specific manner. We use our predictions of the VEGF distribution to quantify a tip cell's receptor binding and gradient sensing capacity. A novel prediction is that neuropilin-1, despite functioning as a coreceptor to VEGF_165_-VEGFR2 binding, reduces the ability of a cell to gauge the relative steepness of the VEGF distribution. Comparing our model to available in vivo vascular patterning data suggests that vascular phenotypes are most consistently predicted at short range by the soluble fraction of the VEGF distributions, or at longer range by matrix-bound VEGF detected in a filopodia-dependent manner.

**Conclusions:**

Isoform-specific VEGF degradation provides a possible explanation for numerous examples of isoform specificity in VEGF patterning and examples of proteases relocation of VEGF upon release.

## Background

Vascular endothelial growth factor A (henceforth called VEGF) is a critical pro-angiogenic factor secreted as numerous splice isoforms that together regulate the phenotype and efficacy of growing vascular networks [1-7]. While the specific mechanism of this control is not fully understood, both isoform-specific receptor binding at the endothelial cell surface [8,9] and differences in the isoforms' spatial patterning [6,7,10-13] are thought to be key. We have previously published studies of the impact of isoform-specific receptor binding [8,14], and here we focus on the spatial patterning. The spatial distribution of VEGF isoforms is thought to be mediated by their interactions with heparan sulfate proteoglycans (HSPGs) in the extracellular matrix (ECM), and by proteases such as the matrix metalloproteinases (MMPs), which can cleave both VEGF [7] and the ECM [15]. Proteases have been shown to have important roles in inducing VEGF-mediated angiogenesis and tumorigenesis [7,16-20]. While it may seem intuitive that ECM binding regulates VEGF diffusion, computational studies suggest that at steady state, simple sequestration by HSPGs may have little effect on the soluble VEGF distribution. The specific mechanisms by which HSPG binding and proteolytic release regulate VEGF diffusion *in vivo *are therefore not yet fully understood, and we explore this here.

**Figure 1:**
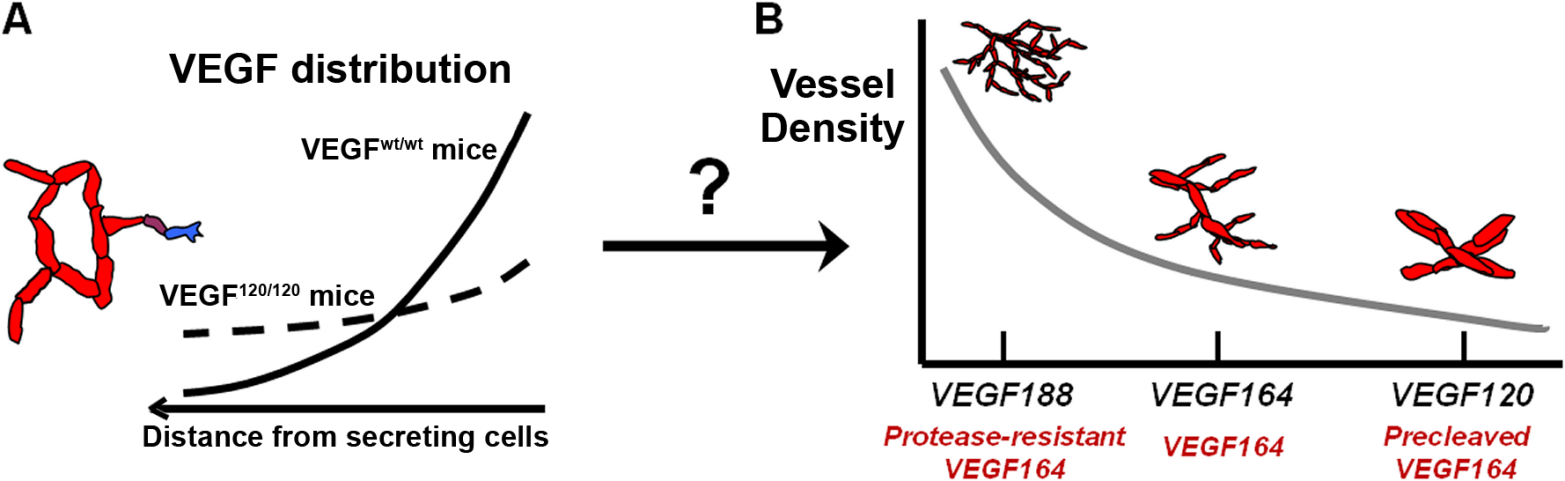
**VEGF isoform patterning and its effects on vascular patterning**. VEGF is patterned in tissues in an isoform-dependent manner, with loss of heparin binding affinity translating to less localized, broader distributions [12,13]. **A**, Isoform specificity of VEGF distribution in the developing mouse hindbrain for mice transfected to express VEGF_120 _versus wildtype mice, which express predominantly VEGF_164 _[94]. **B**, Differences in VEGF localization are thought to give rise to vascular phenotypes that vary with the VEGF isoform, in a monotonic fashion. Larger isoforms, i.e. greater heparin binding affinity, give rise to sprouts that have greater filopodial directionality and vessels with greater branching density and smaller diameters. Similarly for VEGF proteolysis (red text): in tumors, loss of proteolysis exhibits similar features as VEGF_188_-secreting tumors, while VEGF_120_-secreting tumors have similar features as tumors secreting precleaved VEGF [6,7].

In mice, VEGF is primarily secreted as VEGF_120_, VEGF_164_, and VEGF_188 _(human VEGF is one amino acid longer: VEGF_121_, VEGF_165_, VEGF_189_) [3]; longer isoforms include C-terminal motifs that increase binding to heparin and HSPGs in the ECM [21,22]. This increased matrix affinity can reduce the effective diffusivity of the isoform, altering the spatial gradient. For example, transgenic mice expressing only VEGF_120_, an isoform lacking HSPG affinity [23], show a shallow VEGF gradient in the developing hindbrain [13] and retina [12], whereas wildtype mice, which predominantly express the heparin-binding VEGF_164_, have a VEGF spatial distribution that is markedly more localized (Figure 1A). Systems secreting VEGF_188 _show the greatest levels of ECM and basement membrane VEGF deposition [6,24].

As noted above, VEGF sensed by endothelial VEGF receptors has two isoform-specific components: the spatial differences between VEGF isoforms; and the isoform specificity of binding to the receptors themselves as discussed below. These give rise to a spectrum of isoform-dependent vascular phenotypes (Figure 1B). VEGF regulates sprouting angiogenesis at the level of tip cell filopodial guidance and migration and stalk cell proliferation [12]. Systems (mice or tumor xenografts) secreting only the diffusible VEGF_120 _usually develop wide, tortuous, malformed vessels with infrequent branching (Figure 1B) [6,12,13]. This phenotype suggests excessive proliferation and an insufficiency in sprout guidance. On the other hand, VEGF_188 _alone gives rise to vessels that are thin and numerous and have high branching density [3,6]. VEGF_164_, similar to wild type mice, displays intermediate behaviour [4,6,13,25]. While these behaviours are thought to be primarily dependent upon the receptor tyrosine kinases VEGFR2 (KDR/Flk-1) [12], VEGFR1 (Flt-1) also seems to play an important but not yet fully understood role, in part by modulating VEGFR2 signaling [26]. VEGF isoforms also differentially bind to neuropilin-1 and -2 (NRP1 and NRP2), a class of semaphorin coreceptors that function as coreceptors for VEGFR2. Neuropilin-1, for example, can greatly enhance the signaling of VEGF_165 _relative to VEGF_121 _[23,27].

Proteolytic release of VEGF from the matrix seems to biologically mimic secretion of shorter isoforms (Figure 1B). For example, VEGF_120 _elicits vascular effects at greater distances away from the source of secretion than VEGF_164 _[6,12]; similarly, VEGF release by the protease MMP9 enhances VEGFR2 binding in quiescent vasculature by increasing soluble (diffusible) VEGF levels [16], activating tumorigenesis through the angiogenic switch [16,18,19]. Tumor xenografts expressing only the cleaved isoform VEGF_113 _have large dilated vessels [7]. Inhibiting MMP9 results in markedly localized VEGF distributions reminiscent of VEGF_164 _or VEGF_188_, with higher levels of matrix-bound VEGF [6,13,19]. Secretion of a synthetic VEGF_164 _that is resistant to proteolysis (VEGF_164Δ108-118_) induces vascular patterning phenotypes similar to that of VEGF_188 _alone, specifically high vascular densities [7] (Figure 1B). While the direct cleavage of VEGF's C-terminal heparin-binding and NRP1-binding domain is the most accepted mechanism of release (plasmin produces VEGF_110 _from VEGF_165 _[23]; MMP3 produces VEGF_113 _from VEGF_164 _[7]), cleavage of HSPGs and ECM by proteases or heparinases is also a potent release mechanism [15,28-30], but one that liberates intact VEGF. Recently, degradation of soluble VEGF inhibitors such as connective tissue growth factor and soluble VEGFR1 (sVEGFR1) has also been suggested to regulate VEGF [31-33].

The effects of protease-mediated VEGF release are not fully understood. VEGF_164Δ108-118_- and VEGF_188_-expressing tumors show similar intratumoral vessel architectures; however, paradoxically, their ability to enhance tumor growth are vastly different: VEGF_188 _either significantly delays tumor growth [25] or cannot support it at all [6], while VEGF_164Δ108-118 _results in tumor hyperproliferation [7]. Similarly, while most studies implicate MMPs, e.g. MMP9, in pro-angiogenic and carcinogenic behaviors through VEGF [15,16,20,29,32,33], in others, VEGF proteolysis results in an angiogenic response that is uncoordinated and ineffective [17], leading to lower vessel density and decreased tumor growth [7].

It is not fully understood how the spatial patterning of different VEGF isoforms and the processing of VEGF by proteases control endothelial behavior, however several possibilities have been suggested by experiments [7,12,34]. For example, increased matrix-bound VEGF in tissues (due to expression of longer isoforms or inhibition of proteolysis) may increase branching behavior and decrease vessel diameters by increasing p38/MAPK signaling [7,35]. Alternatively, low levels of soluble VEGF may independently increase sprouting behavior [36]. On the other hand, vascular patterning might arise due to neuropilin-1-dependent activation of p38/MAPK signaling [34], supported by the high affinity of VEGF_165 _and VEGF_189 _for NRP1 [37]. Finally, steep VEGF isoform gradients may improve tip cell filopodial stability while mitigating stalk cell proliferation [12].

Whatever the dominant signaling modality that guides vascular patterning, it appears from experimental data to have two particular properties. First, the seemingly monotonic increase in vascular density and decrease in vessel diameter with VEGF isoform length (Figure 1B) suggests that the critical feature of the VEGF distribution that controls vascular behavior also varies in a monotonic fashion. Monotonicity is also evident in the similar vascular phenotypes arising from mice dually expressing VEGF_120 _and VEGF_188_, and those only expressing VEGF_164_, at the same total rate [6,13]. Second, increased VEGF cleavage has the opposite effect to increased HSPG binding [6,7] (Figure 1A). All of the above modalities seem to support both conditions; for example, among the isoforms, VEGF_188 _would be expected to have the highest levels of matrix-bound VEGF [24], lowest levels of soluble VEGF [21,22], most directional gradients [13], and possibly greatest NRP1 affinity [37]. Similarly, VEGF cleavage prevents VEGF binding to HSPGs and NRP1 [23], and by solubilizing VEGF should lead to greater diffusion.

To separate out the above effects, we seek to develop a precise understanding of the effects of HSPG binding and VEGF proteolysis on the VEGF distribution by creating a computational model. The model should replicate key experimental observations regarding the VEGF distribution (summarized in Table 1): longer isoforms with stronger heparin binding affinity have higher degrees of localization (shorter propagation distances and higher local peaks) (Figure 1A) [13]; increased heparin binding affinity results in greater levels of matrix deposition and decreased levels of soluble-phase VEGF [7,21,22,24,38]; and VEGF-cleaving proteases should reverse these patterns [7,16,19]. The inverse relationship between soluble and matrix-bound VEGF gives rise to the question of whether total VEGF is conserved *in vivo*. While this is expected *in vitro *[22,39], and was also seen by certain studies *in vivo *[7,19], other studies found significantly higher total VEGF levels for VEGF_165_-expressing systems [25,39].

**Table 1:** Goals of computational model to match with experimental observations.

VEGF Metric	Effect of a decrease in isoform length	Effect of VEGF cleavage
Level of Soluble VEGF	↑ [21]	↑ [7]
Level of Bound VEGF	↓ [24]	↓ [19] *
Level of Total VEGF	= [7] *	= [7,19] *
Magnitude of soluble VEGF gradients	↓ [13]	↓ [17]
Spatial Range of soluble VEGF	↑ [12]	↑ [17]

Note the concordance of the outcomes of decreased isoform length and VEGF cleavage.

* hypothesized, or some supporting evidence, but no definitive experimental confirmation

The current study extends our work on understanding the nature of VEGF transport in angiogenesis [40-44]. Among the numerous mechanisms of proteases modulating VEGF, we focus on VEGF cleavage, which we have previously studied [44]. Our model describes a sprout situated in a VEGF concentration gradient, to represent a range of biological systems, e.g. the hypoxic front of the hindbrain, tumor, or retina.

**Figure 2:**
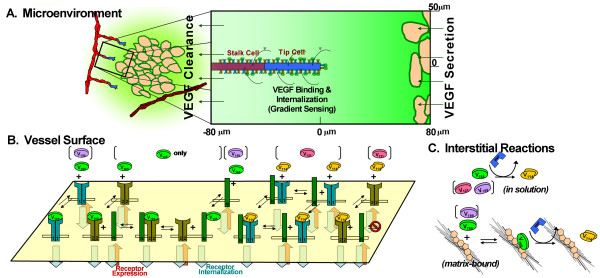
**Schematic diagram of computational model**. We simulate the transport of VEGF in an idealized microenvironment around a sprouting vessel. The above diagram, the base model, includes the processes of VEGF secretion and clearance (**A**), VEGF binding to sprout surface receptors (**B**), and VEGF binding to HSPGs and VEGF proteolysis in the interstitium (**C**). The domain is an axially-symmetric tissue cylinder, 160 μm in length and 50 μm in radius, selected to mimic *in vivo *sprout spacing. The model considers the isoforms VEGF_121_, VEGF_165_, and VEGF_189 _in isolation to compare to single-isoform-expressing *in vivo *systems, and allows for cleavage of isoforms into VEGF_114_, the MMP-cleavage product. Receptor signaling is modeled with the isoforms' interactions with VEGFR1, VEGFR2, and NRP1 (B). While the reactions are shown primarily for VEGF_165_, isoforms VEGF_121 _and VEGF_189_, where bracketed, participate in similar reactions. VEGF and receptor-binding gradients are measured between the front and rear of the tip cell (indicated in gray arrowheads) (A). Extensions of this model explored here include gradients of HSPG and VEGF degradation (Figure 4).

We use the computational model (Figure 2) to understand mechanisms that might allow isoform-dependent VEGF localization and proteolytic redistribution to arise. Using a VEGF-VEGFR kinetic binding model, we investigate how VEGF signals may be detected by endothelial cells, e.g. soluble or matrix-bound VEGF, and the impact of NRP1. The results suggest that the system must exhibit isoform specificity in VEGF clearance or degradation: along with longer isoforms binding to the ECM with greater affinity, the longer isoforms must also be cleared or degraded from the soluble fraction at faster rates.

## Methods

### Model Formulation

To study the role of different isoforms and VEGF proteolysis on the VEGF distribution, we constructed a 3D reaction-diffusion-based computational model of VEGF transport. The model is centered on a capillary sprout containing a single endothelial tip cell followed by stalk cells (Figure 2A), to study the VEGF concentration profile and gradients in the vicinity of the sprout, and to understand the receptor signaling and gradients detected by VEGF receptors (primarily VEGFR2) on the endothelial tip cell. To dissect the behavior of individual isoforms, we consider separate systems expressing a single isoform in isolation, similar to those tested experimentally in developmental and tumor models.

A schematic of the VEGF transport and reaction is presented in Figure 2B. In our model, we label our VEGF using the human isoforms nomenclature and represent cleaved VEGF by VEGF_114_, which is the predicted MMP-cleavage product of human VEGF [7]. While Figure 2 primarily details the interactions of secreted VEGF_165_, we also separately consider VEGF_121 _and VEGF_189_, and hypothetical isoforms of intermediate lengths and properties. These intermediate isoforms are generated by smoothly varying the relevant parameters: HSPG affinity, NRP1 affinity, and ability to bind the VEGFR1-NRP1 complex. While VEGF_121 _has weak affinity to NRP1 [45], we assume it does not bind NRP1, and this does not change our results [43]. Intact (unproteolysed) VEGF_189 _may be unable to bind VEGFR2 [46], but binding of long isoforms of VEGF to VEGFR2 is predicted to be low in our model due to degradation and cleavage; cleaved VEGF represents the majority of VEGFR2 binding in this case.

**Figure 3:**
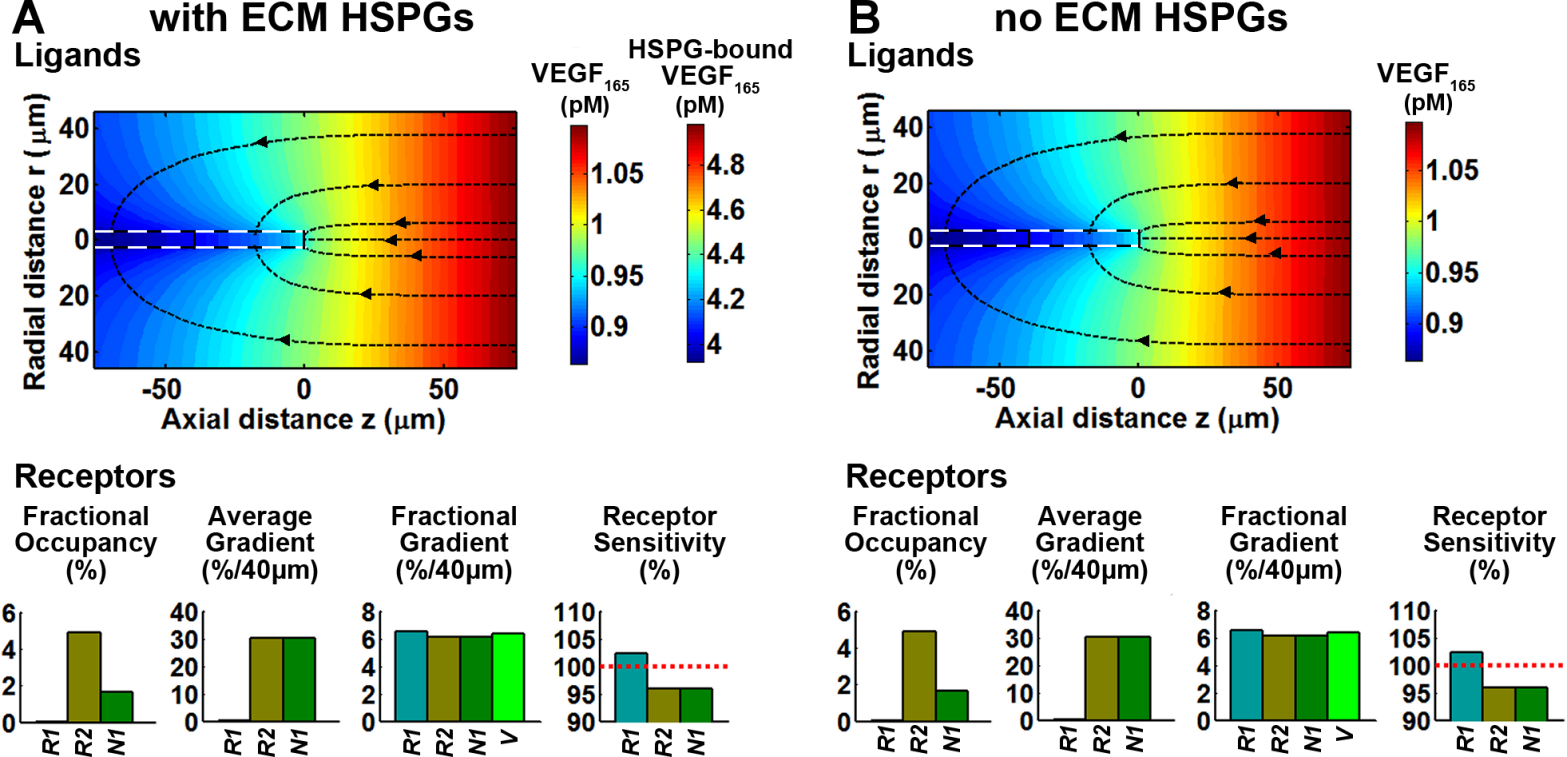
**Steady-state distribution of VEGF is not dependent upon interstitial HSPGs**. Using the model, we calculated the steady-state distribution of VEGF_165_, in the presence (**A**) and absence (**B**) of interstitial HSPGs, assuming no interstitial proteases, and no other differences between the two systems. The VEGF distribution was initialized so as to have a mean soluble VEGF concentration of ~1 pM and a fractional gradient of ~5%/40 μm at the leading edge of the sprout (z = 0); HSPG binding and receptor-mediated internalized were calculated. Conditions of the model are defined in Table 4: HSPG = 750 nM, VEGFR1 = VEGFR2 = 10^4^/cell, NRP1 = 3*10^4^/cell; secretion and clearance of the soluble fraction occurred at q = 5.27·10^-5 ^molec/μm^2^·sec and k_clear _= 0.0399 μm/sec. Traces of steepest descent arising from r = 0, 6, 20, and 38 μm and z = 80 μm are overlaid to demonstrate the effect of internalization on the curvature of the VEGF distribution around the sprout. We also measured VEGFR1, VEGFR2, and NRP1 receptor occupancies, absolute and fractional gradients, and the sensitivity of relative gradient detection are given for each case. The sensitivity is defined as the ratio of the receptor gradient to the ligand gradient.

We assume that VEGF is secreted from the leading face of the cylindrical domain and is cleared by two mechanisms: internalization by the sprout's VEGF receptors; and clearance at the trailing edge of the domain (which physiologically represents the effects of both diffusion and internalization by downstream vasculature). As a base model (Figure 2, 3), we consider that the sprout expresses all three VEGF receptors, in a combination where excess NRP1 plays a potentiating role for VEGF_165 _binding to VEGFR2 [27]. The sprout is situated in a cylindrical domain consisting of an acellular extracellular matrix (ECM) that contains uniform HSPG binding sites for VEGF. For VEGF proteolysis, we assume a uniform, single-step irreversible first-order MMP-mediated cleavage of VEGF to VEGF_114 _(developed previously in [44]). The dynamics of protease activation and inhibition have been studied in the past [47-49], but are neglected here because of uncertainties in the transport of the protease itself.

We have previously shown that the thin basement membrane layer does not significantly affect VEGF diffusion near the sprout [44], and thus do not consider the basement membrane here. As in previous models [41,42,50], receptors are considered pre-dimerized and binding of the ligand is a reversible single-step reaction; in addition, internalization and receptor insertion are balanced to keep plasma membrane receptor levels constant. Finally, though matrix-bound VEGF may be relevant to VEGFR2 activation [35], our model only considers receptors binding to soluble VEGF.

We study several specific models with increasing complexity, to understand the mechanisms behind gradient formation. The model components as described above - diffusion, receptor binding, proteolytic processing - comprise the "HSPG-binding-only" model. This is so named to contrast with other models that add additional processes relevant to VEGF patterning in vivo: patterned HSPG gradients (the "HSPG-gradient" model) and VEGF degradation. VEGF degradation is defined here as any interstitial process that destroys VEGF activity, without generating an active cleavage fragment as proteolysis does. As with proteolysis, degradation was characterized by a first-order rate constant, k_deg_. The study of degradation was done with two models: one assuming that degradation affects only soluble VEGF (the "soluble VEGF degradation" model); and one in which both soluble and matrix-bound VEGF are degraded (the "matrix-sequestered VEGF degradation" model). We will see later that the soluble VEGF degradation model, in which HSPG-bound VEGF is protected from degradation, is effectively isoform-independent degradation; while degradation of HSPG-bound VEGF results in isoform-dependent degradation. Longer isoforms have higher degradation (despite having the same rate constant) due to longer retention times.

In studying receptor binding, we incorporate a sprout expressing VEGFR1, VEGFR2, and NRP1. This sprout can itself influence VEGF transport (by receptor-mediated uptake), and to study the patterning of VEGF that would arise only by diffusion, matrix binding, proteolysis and degradation, we also considered a variant of the model that contains no sprout.

### VEGF/Protease Transport and Reactions

VEGF biochemical reactions and transport processes are described by partial differential equations with appropriate boundary conditions. The computational domain consists of two components: the interstitium, into which VEGF is secreted, and a sprout surface layer, on which VEGF capture by the receptors is modeled. Upon secretion from the leading edge of the domain (z = +L), VEGF transport within the domain is governed by the mass balance (VEGF_165 _equation shown; similar equations for other isoforms):(1)

Here [V_165_] describes the volumetric "bulk" concentration of the soluble fraction of VEGF_165_, assuming an isotropic porous matrix with available volume fraction K_ECM_. D_165 _is the effective diffusion coefficient for VEGF_165 _in the matrix; [H] is the density of VEGF-binding sites, with reaction rate constants, k_on_^V,H ^and k_off_^V,H^. k_P _describes first-order net rate of proteolysis, and k_deg _describes first-order degradation of soluble VEGF. We assume that k_P _and k_deg _are spatially uniform. The values of these parameters are given in Table 2 and described further below.

**Table 2:** Kinetic Parameters

Isoforms Involved	Reaction	Forward k_on_, k_c_, k_int_	Reverse k_off_, k_uc_	K_eq_	**Ref**.
	***Cell-surface Reactions***				
121, 165	Binding VEGF to VEGFR2	1·10^7 ^M^-1^s^-1^	0.001 s^-1^	100 pM	[42]
All	Binding VEGF to VEGFR1	3·10^7 ^M^-1^s^-1^	0.001 s^-1^	33.3 pM	[42]
165, 189	Binding VEGF to NRP1	3.2·10^6 ^M^-1^s^-1^	0.001 s^-1^	312 pM	[42]
165	Binding VEGF_165_-VEGFR2 to NRP1	3.1·10^6^ [10^15 ^μm^2^/(mol·s)]	0.001 s^-1^	323 mol/10^15 ^μm^2^	[42]
165	Coupling VEGF_165_-NRP1 to VEGFR2	1·10^7^ [10^15 ^μm^2^/(mol·s)]	0.001 s^-1^	100 mol/10^15 ^μm^2^	[42]
121, Cleaved	Coupling VEGFR1 to NRP1 or Coupling VEGF_114_-VEGFR1 to NRP1	1·10^7^ [10^15 ^μm^2^/(mol·s)]	0.01 s^-1^	1000 mol/10^15 ^μm^2^	[42]
-	Internalization, k_int_	2.8·10^-4 ^s^-1^	-	-	[42]
-	Insertion, s_R2_, s_R1_, or s_N1_	e.g., k_int._·[R2]^total^	-	-	calculated
					
	***ECM Reactions***				
165	VEGF_165 _to HSPG	6.06·10^4 ^M^-1^s^-1^	0.01 s^-1^	165 nM	[63]
189	VEGF_189 _to HSPG	1.18·10^6 ^M^-1^s^-1^	0.01 s^-1^	8.5 nM	Figure S1.1
All	Degradation rate, k_deg _(only Figs. 4C,D; 8A)	0.001 s^-1^	-	-	assumed
All	Protease activity, k_P _(only Figs. 6; 7A; 8A)	2.8·10^-4 ^s^-1^	-	-	assumed

Cleaved VEGF (VEGF_114_) is generated from the proteolysis of free VEGF or matrix-bound VEGF (Figure 2C).(2)

VEGF proteolysis also produces an HSPG-binding C-terminal fragment [23] that maintains heparin affinity, but we do not include it explicitly as HSPGs are not saturated (i.e. [V]<< K_d_^V,H^). The matrix components, HSPG and VEGF-HSPG do not diffuse:(3)(4)

### Boundary conditions for VEGF reaction at the cell surface

VEGF and cleaved VEGF are coupled to the cell-surface VEGF receptor population through the specification of boundary conditions. These boundary conditions equate the flux of VEGF perpendicular to the cell surface, J = -D·∂V/∂n, with the rate of reaction at the cell surface. For VEGF_165 _and VEGF_189_, these reactions include binding to VEGFR2, VEGFR1, and NRP1, while for VEGF_114 _and VEGF_121_, binding occurs to VEGFR2, VEGFR1, and the VEGFR1-NRP1 complex (rate parameters for the individual isoforms are given in Table 2).(5)(6)

Equations describing the VEGF receptors are given as a system of ordinary differential equations over the sprout surface. In addition to describing VEGF binding to the receptors, they describe the continual balance between secretion of unligated VEGF receptors (at a rate s_R_) and internalization (k_int_), VEGF_165 _bridging of VEGFR2 and NRP1, VEGFR1/NRP1 coupling (k_c_) and uncoupling (k_uc_), and VEGF_114 _binding to the VEGFR1-NRP1 complex. Note that VEGF_121 _and VEGF_189 _terms are omitted for clarity. We assume that receptors do not diffuse along the cell surface.(7)(8)(9)(10)(11)(12)(13)(14)(15)(16)(17)

### External Boundary conditions for VEGF secretion and clearance

The computational domain is assumed to be representative of its surrounding tissue in the radial direction. Thus, we use a no-flux condition, ∂V/∂r = 0 at r = R_edge_. The VEGF gradient was specified by secretion of uncleaved VEGF at z = +L using the Neumann BC, -D_V_·∂[V]/∂z = -q, and a first-order VEGF clearance at z = -L, -D_V_·∂[V]/∂z = -k_clear_[V]. The secretion rate q and k_clear _were pre-calculated to generate the desired VEGF distribution in the absence of proteases, i.e. a concentration V_0 _at z = 0 and a VEGF gradient, g_0 _over the domain length. For cleaved VEGF, there was no secretion, but there was clearance. Protease activity and degradation were assumed to be uniform.

The volume-averaged clearance rate of soluble VEGF in our model, k_clear_/(2L) ~ 2.49·10^-4 ^s^-1^, is similar in magnitude to clearance times *in vivo *[51] and represents internalization by downstream cells and transvascular permeability.

### Steady-state assumption and consequences

The steady-state approximation assumed in our simulations is justified because VEGF diffusion and reaction, which our model considers, occur much more rapidly than the structural changes in vessels and parenchymal cells, which may take days to weeks. Transients in the VEGF distribution are limited by the residence time of VEGF in tissue, which has been experimentally [51-53] and theoretically [42] shown to be ~1 h or less. The steady nature of the VEGF distribution has been experimentally confirmed in the hindbrain, where the VEGF distribution is relatively unchanged over an 18 h window [13] and still maintains isoform specificity. If transient diffusion does contribute to the specificity of VEGF gradients, we would expect vascular patterning to lose its isoform-dependence over long periods, which is not seen either in the retina (1 week [12]) or in tumors (several weeks [6,7,19]). The same may be true for in vitro and ex vivo systems (1-3 days, [7,54]).

The VEGF transport equations can be simplified at steady state. We perform the analysis for VEGF_165_, but the analysis is applicable to any isoform. In the absence of proteases, VEGF is in equilibrium with HSPG (k_on_[V][H] = k_off_[VH]) thus the VEGF diffusion equation reduces to:(20)

This equation states that VEGF transport is not influenced by HSPG binding. In the presence of proteases, VEGF_165 _can be converted to VEGF_114_, and summation of the VEGF_165 _and VEGF_114 _equations and neglecting minor variations in the intrinsic diffusivity of the two isoforms, leads to:(21)

Thus, the total soluble VEGF is specified by the same equation regardless of the presence or distribution of VEGF-cleaving proteases. If receptor-mediated internalization is identical between the isoforms (e.g. in the absence of NRP1), total soluble VEGF should not be altered by proteases.

Another important quantity, matrix-bound VEGF, can be calculated using the relation, [H] = [H]_Total _- [VH]. Assuming a uniform protease distribution, with proteolysis rate k_P_, we obtain(22)

Note that for typical parameters used in this study, k_off _>> k_P _>> k_on_[V], we can approximate VEGF/HSPG binding by [VH] ~ [V][H]_Total_/K_d _(i.e. that [VH] is directly proportional to [V] and total HSPG). The steady-state distribution of the soluble fraction of VEGF can thus be described by:(23)

Thus, VEGF proteolysis is enhanced not only by the rate of proteolysis, k_P_, but also by matrix binding, which decreases VEGF's effective diffusivity, D_Eff _= D/(1 + [H]_Total_/K_d_). As a result, matrix binding potentiates VEGF proteolysis.

### Numerical Methods for VEGF Calculations

The transport of VEGF and the dynamics of HSPG were solved using the finite volume method in cylindrical coordinates (z and r), Figure 2. The control volume spacing in the z direction was 8 μm. In the r direction, one voxel was used to represent the sprout radius, from r = 0 to r = R_sprout_; for r ≥ R_sprout _(outside the sprout surface), spacing was 4-8 μm. A finer grid produces consistent results different by less than 5% and does not alter qualitative conclusions. Heterogeneous reactions at the cell surface were approximated as homogenous reactions, using a basement membrane layer formulation given previously [40].

The steady-state solution was obtained by solving the transient solution from the initial conditions until a relative convergence of 10^-7 ^was achieved at each node. First-order temporal derivatives were discretized using a first order fully-implicit scheme, while the second-order spatial derivatives were discretized using a second order central difference method. Nonlinear solution of the equations was found by iteration using the successive over-relaxation (SOR) update formulation and a Red-Black node ordering [55]. Simulations were run on a personal computer using custom code written for Matlab 7.6.0.

### Model Implementation and Initial Conditions

Because the cellular internalization of VEGF creates a region of VEGF depletion near the sprout, to be consistent we ran each simulation with an initial VEGF gradient in the absence of proteases or degradation. We first imposed Dirchelet BCs satisfying the overall domain gradient of VEGF, g_0_, and mean VEGF concentration V_0 _at z = 0 (see *Parameters*): at z = +L, [V_165_] = V_0_·(1+g_0_·L/L_tip_), at z = -L, [V_165_] = V_0_·(1-g_0_·L/L_tip_)), setting any degradation and proteolysis rates to be zero. This simulation can be done in the absence of HSPGs to reach convergence more quickly since HSPGs have no influence on steady-state soluble VEGF under these conditions (see previous section). After equilibration of diffusion and the VEGF receptors, Dirichlet BCs were converted into Neumann BCs specified by q and k_clear_, defined using the following formulas:(18)(19)

Due to averaging over the respective boundaries, these equations only approximately specify the conditions, g_0 _and V_0_; however this is sufficient for our study. After this step, we incorporated HSPGs and VEGF-HSPG complexes at equilibrium, any degradation terms and proteolytic reactions to arrive at the predicted steady-state spatial distribution.

### Geometrical and Transport Parameters

The tissue (cylindrical computational domain) is 160 μm long (L = 80 μm) and 100 μm in diameter (R_edge _= 50 μm) (Table 3, Figure 2A). The sprout is a cylinder from z = -80 μm to z = 0 with a radius of R_sprout _= 2 μm. These dimensions were chosen to reflect the average sprout size and sprout-to-sprout distance based on micrographs of sprouting vasculature [12,13,56].

**Table 3:** Physical and Transport Parameters

Parameter	Value	**Ref**.
Radius of domain, R	50 μm	Assumed
Half-Length of domain, L	80 μm	Assumed
Length of tip cell, L_cell_	40 μm	[12]
Radius of sprout, R_sprout_	2 μm	[12]
	Tip cell surface area: 515 μm^2^	calculated
Diffusivities, D_114, _D_121, _D_165_, D_189_	68.6 μm^2^/s (see *Methods*)	estimated
Local Available Volume Fraction of the extracellular matrix, K_ECM_	0.85	[57]

The matrix composition affects both the diffusivity and the available volume for VEGF transport. We estimate the available volume fraction of the ECM as 0.85 consistent with experimental data on transport studies of dextran in cellularized agarose gels [57]. Diffusivity was calculated by estimation of the aqueous diffusivity, D_aq_, at 37°C using data from Berk et al. and the Stokes-Einstein relation [58,59]. The hindrance of the matrix was then approximated using Ogston's relation, assuming a matrix composition of 14% v/v collagen fibrils (r_h _= 20 nm), 0.078% v/v GAG chains (r_h _= 0.55 nm) as in [60]. Finally, we corrected for the increased viscosity due to proteinacious interstitial solution (20.6 g protein/L) [61], resulting in D_165 _= 68.6 μm^2^/s. Despite a slight error, we standardized the results of the different VEGF isoforms by assuming D_165 _for also VEGF_114_, VEGF_121_, and VEGF_189_. Receptor movement is neglected as diffusivity along the cell surface, D ~ 0.01 μm^2^/s [62] is much slower than reactions at the cell surface; the Damköhler number is Da = k_off_·L_tip_^2^/D = 160.

### Kinetic Parameters for Reactions

To estimate the binding of VEGF_165 _to HSPGs, we used binding rates of bFGF to HSPGs (k_on _= 4.2·10^5 ^M^-1^s^-1^, k_off _= 0.01 s^-1^, K_d _= 23.8 nM) [63] and adjusted k_on _to match K_d _estimates of VEGF_165 _binding to heparin, K_d _~ 165 nM [38,64]. VEGF_189 _binds to heparin and HSPGs more strongly than does VEGF_165_, however no estimate for its K_d _had been made. Based on extrapolation of VEGF_165 _heparin elution data (described in more detail in Additional file 1, *section S1*; *Figure S1.1B*), we estimated a lower limit for K_d _of 8.5 nM. VEGF_121 _and VEGF_114 _do not bind to HSPG [23]. The overall rate of VEGF cleavage by proteases in tissues is not known; but we assumed a rate that produces significant effects for our model parameters, k_P _= 2.8·10^-4 ^s^-1 ^(time-scale of 1 hr), which is similar to VEGF clearance rates *in vivo*.

We previously characterized kinetic parameters for the binding of VEGF to VEGFR1, VEGFR2, and NRP1, and for the coupling of the receptors (see Table 2) [42]. While VEGF_165 _has several fold greater affinity for VEGFR2 (in the presence of HSPG) than VEGF_121 _[23,38,65] and VEGF_121 _may bind NRP1 with low affinity [45], we assume that the differences in the isoform binding to receptors are due to differences in NRP1 binding: no binding for VEGF_121_; affinity for VEGF_189 _higher than that of VEGF_165 _in proportion to the isoforms' HSPG affinity. The behavior of cleaved VEGF_110 _(and presumably VEGF_114_) has been shown to be identical to that of VEGF_121 _[23] indicating similar receptor kinetics to VEGF_121_. Finally, we assume receptors are internalized at a rate, k_int_, independent of VEGF binding [66] and that each VEGFR is maintained at a constant level, i.e. s_R2 _= k_int_·[R2]_Total_.

As described above, we studied a hypothetical continuum of VEGF isoforms with properties intermediate between the three isoforms: we varied VEGF receptor binding parameters along this continuum proportionally to the isoforms' affinity for HSPGs. Two opposing effects needed to be accounted for: the ability of VEGF to couple VEGFR2 and NRP1 and the ability of VEGF to bind to the VEGFR1-NRP1 complex. Thus the VEGF-NRP1 binding rate was k_on_^V,N1 ^= k_on_^165,N1^·(K_d_^165,H^/K_d_^V,H^). The coupling rate, k_c_, between VEGF-VEGFR2 and NRP1 was also increased by the same factor to preserve equilibrium. In contrast, VEGF binding to the VEGFR1-NRP1 complex and NRP1 coupling to the VEGF-VEGFR1 complex was higher for shorter isoforms, with k'_forward _= k_forward_·(K_d_^V,H ^- K_d_^165,H^)/K_d_^165,H ^and k_reverse _unchanged, for isoform HSPG binding affinities greater than K_d_^165,H ^= 165 nM (any isoform with greater affinity to HSPGs than VEGF_165 _has no binding to VEGFR1-NRP1).

A degradation rate constant of k_deg _= 10^-3 ^s^-1 ^was used to estimate the impact of significant degradation. As we will see, total degradation is independent of the isoform if the HSPG-bound isoforms are protected against degradation. Conversely, degradation is isoform-specific when the heavier isoforms can be degraded when bound to HSPGs as well as in solution. The heavier isoforms will be degraded more due to their longer residence time.

### Concentrations of Receptors and HSPG

Concentrations of VEGF, abluminal VEGF receptors, and ECM HSPG were estimated previously [8,42,60,67] (see Table 4). VEGF levels were taken to be V_0 _= 1 pM as previously determined from the literature [67]. We assume that each endothelial cell has a fixed population of VEGFR maintained by a balance of receptor insertion into the plasma membrane and internalization. We assumed a base condition of [R1]_Total _= 10^4 ^and [R2]_Total _= 10^4 ^per vascular surface corresponding to the area of a tip cell (515 μm^2^). To account for the excess of NRP1, we use [N1]_Total _= 3·10^4 ^molecules/515 μm^2^. The binding site density of HSPG in the ECM was 750 nM [41].

**Table 4:** Model Conditions

Parameter	Symbol	Value	**Ref**.
Initial mean VEGF level	V_0_	1 pM	[67]
VEGF gradient over domain	g_0_	5%/40 μm	Assumed
[HSPG] in ECM	[H] _Total_	0.75 ·10^-6 ^mol/L	[41]
*System with receptors*			
Total VEGFR2 levels per area of tip cell on vasculature	[R2]_Total_	10,000/(area of tip cell); area of tip cell = 515 μm^2^	[42]
Total VEGFR1 levels per area of tip cell on vasculature	[R1]_Total_	10,000/(area of tip cell)	Assumed
Total NRP1 levels per area of tip cell on vasculature	[N1]_Total_	30,000/(area of tip cell)	Assumed
VEGF (typically VEGF_165_) secretion rate	q	5.27·10^-5 ^molec/μm^2 ^·s	Calculated
VEGF clearance rate	k_clear_	0.0399 μm/s	Calculated
*System without receptors*			
VEGF (typically VEGF_165_) secretion rate	q	4.39·10^-5 ^molec/μm^2^·s	Calculated
VEGF clearance rate	k_clear_	0.0948 μm/s	Calculated
*HSPG gradient *(Figure 4B)			
HSPG gradient	g_H0_	30%/40 μm	Assumed
*Soluble VEGF degradation *(Figure 4C)			
Degradation rate of soluble VEGF	k_deg_^V^	0.001 s^-1^	Assumed
Degradation rate of matrix-bound VEGF	k_deg_^VH^	0 s^-1^	Assumed
*Matrix-sequestered VEGF degradation scheme *(Figure 4D)			
Degradation rate of soluble VEGF	k_deg_^V^	0.001 s^-1^	Assumed
Degradation rate of matrix-bound VEGF	k_deg_^VH^	0.001 s^-1^	Assumed

### Definition of Gradient Metrics for VEGF isoforms and VEGF receptors

Endothelial cells may sense absolute or relative gradients of VEGF [68,69]. For initiation of vascular sprouting, the gradient across one cell is likely to be sensed. For sprout extension and maintenance of the tip cell/stalk cell differentiation, the signaling difference between two cells (e.g. a tip and stalk cell [70]) may be more relevant.

In the present study we consider gradients only between the front and back of the surface of the tip cell, with mean concentrations ([V]_Tip_) defined over the tip cell's entire surface. The absolute gradient, denoted by AG_V _or Δ_Tip_[V] is the difference in VEGF concentration between the tip cell front and back. Note that the absolute gradient is additive over all isoforms, e.g. AG_V _= AG_165 _+ AG_121 _for a two isoform distribution. The steepness of the VEGF distribution (relative gradient) is the absolute gradient normalized to either the mean tip total soluble VEGF, i.e. fractional gradient or FG_165_, or to the mean tip concentration of the isoform under consideration, e.g. IFG_165_:(24)(25)

Note that the above metrics differ only in the presence of multiple VEGF isoforms.

We use similar definitions to find gradients of cell-surface VEGF-bound receptor complexes. In this case, the gradient is limited to the axial (z) direction due to rotational symmetry in our model. We normalize the absolute gradient of receptor complexes to the total amount of a particular receptor, e.g.:(26)

where [R2]_Total, Tip _is the sum of all forms of VEGFR2 on the tip cell surface. Using the fractional occupancy (FO) of a VEGF isoform to a VEGF receptor, the fractional gradient of VEGF_165 _bound to VEGFR2 in a system consisting of isoforms VEGF_121 _and VEGF_165 _can be expressed as:(27)

We introduce the concept of sensitivity, S, to define the multiplicative relation between a metric in VEGFR2 binding and a similar metric in the VEGF distribution, e.g. S_IFG-165 _= IFG_165-R2_/IFG_165_. Note that S_IFG _also describes the factor increase in the fractional occupancy (FO) given a factor increase in [VEGF], strictly valid only in the differential limit:(28)

For a single receptor detecting a single ligand, S_IFG _is always less than 1 (S_IFG _= 1 - FO) (for more detail, see Additional file 1, *section S2.2*).

### Additional Assumptions

We assume that degradation and proteolysis is uniformly distributed. Our model does not also fully reflect the contribution of vasculature to VEGF transport. While vasculature-mediated internalization is thought to represent the primary clearance for VEGF in tissue [43], our model only implicitly represents such effects (through a clearance at z = -L), neglecting isoform-specific variation. We also assume constant internalization and insertion rates for the VEGF receptors, resulting in constant total receptor population on the sprout. This is justified because we are primarily interested in the sensory role of the sprout and not in its internalization capacity, however ligand-induced shifts in NRP1 and VEGFR2 may also alter sensory function. Mechanistic models of these processes have not been developed and thus we did not incorporate them in the current study.

## Results

### The computational model predicts that the steady-state distribution of soluble VEGF is isoform-independent when considering only diffusion and matrix binding

When VEGF is secreted, extracellular HSPGs are predicted not to influence the distribution of the soluble fraction of VEGF (Figure 3A vs. 3B); nor do they influence receptor signaling (Figure 3, bar graphs), assuming VEGF-HSPG complexes in the ECM cannot directly ligate VEGF receptors. At steady state, each molecule of VEGF that binds to the matrix is matched by a molecule that unbinds. Note that using physiological parameters, VEGF concentration is significantly below the K_d _for matrix binding ([V] ~ 1 pM, K_d _= 165 nM) resulting in HSPG binding sites remaining unsaturated; in this regime, matrix-bound VEGF concentration is linearly proportional to soluble (unbound) VEGF concentration at all spatial positions.

At the tip cell, the absolute gradient of unbound VEGF is ~0.059 pM/40 μm. The direction of the gradient of receptor signaling (characterized in this study by the receptor occupancy) is the same, with magnitude ~30 VEGF-VEGFR2/40 μm. The *relative *steepness (isoform fractional gradient, IFG) of both the VEGF and VEGF-VEGFR2 distributions are similar, IFG_VEGF _= 6.39%/40 μm and IFG_VEGF-VEGFR2 _= 6.12%/40 μm. Thus, the sensitivity of VEGFR2 binding to VEGF gradients (S_IFG_) is high (Figure 3B). Note that VEGF receptors also locally deplete VEGF and thereby influence the diffusion of VEGF (Figure 3, arrowed dotted lines); in the case shown, receptors capture ~39% of the secreted VEGF.

### Differences in VEGF gradients can arise from differences in VEGF degradation

**Figure 4:**
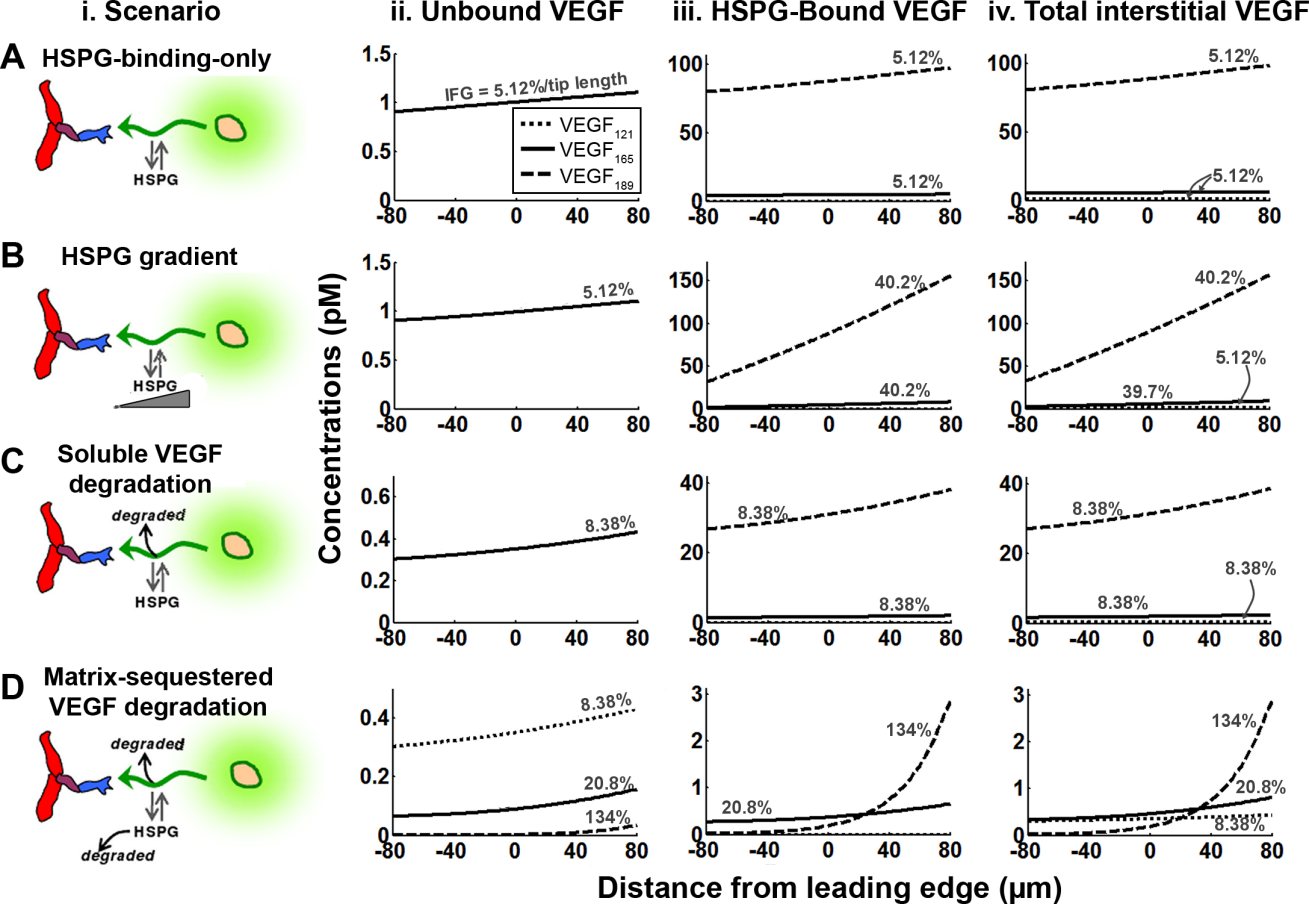
**Mechanisms of forming differential isoform gradients**. We consider the distributions of VEGF_121_, VEGF_165_, and VEGF_189 _under four scenarios that may be responsible for isoform patterning *in vivo *(i, soluble fraction; ii, bound fraction; iii, soluble + bound VEGF). **A**, reversible HSPG binding considered previously in Figure 3 (HSPG-binding-only model). **B**, patterning of the underlying HSPG ([H]_Total _= 750 nM at z = 0, 30%/40 μm). **C**, soluble VEGF degradation; this is isoform-independent degradation (all isoforms are degraded at the same rate), but HSPG-bound VEGF is protected from degradation; k_deg _= 10^-3 ^s^-1^. **D**, matrix-sequestered VEGF degradation; all isoforms are degraded, and HSPG binding confers no protection; this has the effect of increasing degradation of HSPG-binding isoforms due to their longer residence time, even though all isoforms have the same degradation rate constant k_deg _= 10^-3 ^s^-1^.

The lack of influence of HSPGs on VEGF gradients at steady state suggests that different isoforms secreted at the same rate (as occurs in transgenic systems designed to express only one isoform), and differing only in HSPG binding, will have similar soluble VEGF distributions (Figure 4Aii). Matrix-bound VEGF increases with HSPG affinity of the isoform (Figure 4Aiii), as does the total amount of extracellular VEGF (for VEGF_121_: 646 molecules; VEGF_165_: 3.58·10^3 ^molecules; VEGF_189_: 5.76·10^4 ^molecules). However, because bound VEGF is proportional to soluble VEGF, the relative gradients of matrix-bound VEGF are the same for each isoform. Over the simulated length scales, total VEGF concentration gradients of different isoforms would not intersect (Figure 4Aiv), regardless of HSPG concentration (*not shown*). Thus, differential HSPG binding of isoforms is not sufficient to account for observed VEGF patterning in vivo (Figure 1A).

We simulated whether a patterned ECM (Figure 4B) could cause the observed gradients, but this scenario also did not lead to isoform-specific differences in soluble VEGF; however, it did lead to larger isoforms having steeper distributions in bound and total VEGF (Figure 4Biv). Still, VEGF levels between different isoforms are not predicted to intersect within the spatial frame of reference.

We first considered whether the steady-state assumption was accurate, since transient diffusion of isoforms having different HSPG affinities can momentarily lead to differential isoform patterning. However, experimental evidence points to VEGF_121 _and VEGF_165 _having rapid kinetics *in vivo *(τ ~ 1 h) [51,71], which likely indicates that VEGF patterning operates close to steady state. We will consider the ability of VEGF-cleaving proteases to create these spatial distributions; however, it is of note that VEGF fragments have not yet been observed in developing tissues [17].

Next, we considered VEGF degradation (Figure 4C,D). In vivo, VEGF diffuses in an environment filled with cells, which may selectively uptake VEGF isoforms, e.g. through cell surface receptors, possibly in conjunction with NRP1 or cell-surface HS [72]: these effects are incorporated into the VEGF degradation term of our model. Degradation may also result from VEGF inactivation by isoform-selective VEGF inhibitors, e.g. connective tissue growth factor, sVEGFR1, thrombospondin-1, or by proteases that can cleave VEGF to fragments that are not recognized by commonly employed antibodies.

As described in the methods section, we considered two specific cases for degradation: first, the "soluble VEGF degradation model" in which HSPG-bound VEGF is protected from degradation (Figure 4C); and the "matrix-sequestered VEGF degradation" model, in which HSPG-bound VEGF can be degraded (Figure 4D). The former is similar to heparin-bound bFGF being protected against plasmin cleavage [73].

Computational simulations suggest that when HSPGs protect VEGF (Figure 4C), the soluble VEGF distribution is independent of HSPG binding, as if the HSPGs were not even present. The relative gradients of the VEGF isoforms are equal (8.4%/tip length) and thus cannot recapitulate isoform-dependent localization. In addition, total VEGF in the system is approximately proportional to the HSPG binding affinity.

In contrast, when HSPG-bound VEGF is vulnerable to degradation (Figure 4D), the different grading of VEGF isoforms seen in vivo (e.g. Figure 1A) is recapitulated [7,21,22,39]. Specifically, the strongly heparin-binding VEGF_189 _produces a very steep distribution (Figure 4Div). As a consequence of this, close to the source of secretion, increased ECM-binding affinity (i.e. longer isoform) corresponds to increased levels of bound VEGF; but at sufficiently far distances, this trend is reversed and the highly diffusible isoform is more abundant (Figure 4Diii). Despite different spatial localization, the total amount of VEGF in the system is similar for each isoform (for VEGF_121_: 229 molecules; VEGF_165_: 345 molecules; VEGF_189_: 379 molecules), and may be consistent with data from [7,22].

### Proteolytic cleavage of bound VEGF increases soluble VEGF only in a model of isoform-specific degradation

**Figure 5:**
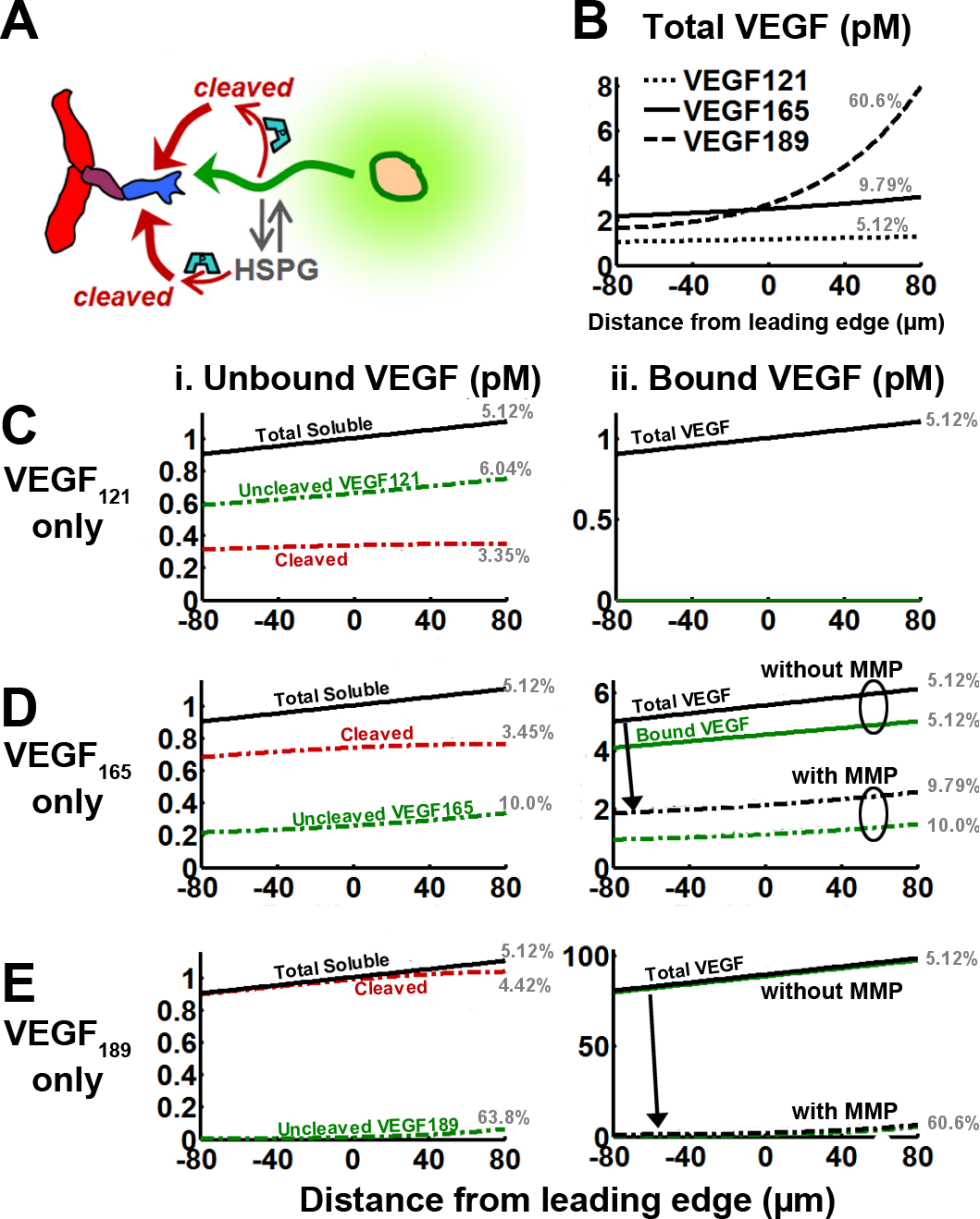
**Effect of VEGF-cleaving MMPs on the VEGF distribution**. Proteases (MMPs) were added to the HSPG-binding-only system at k_P _= 2.8·10^-4 ^s^-1 ^for systems expressing VEGF_121 _(C), VEGF_165 _(D), or VEGF_189 _(E). We look at the components of the soluble VEGF distribution (i), and bound and total VEGF (ii), both before the addition of proteases (solid lines), and after (dashed lines). The total VEGF distributions of different isoforms, in the presence of proteases, are displayed against each other (B). Simulations were performed in the absence of receptors (parameters given in Table 4). Isoform fractional gradient values at the tip cell are noted in grey with units %/40 μm.

**Figure 6:**
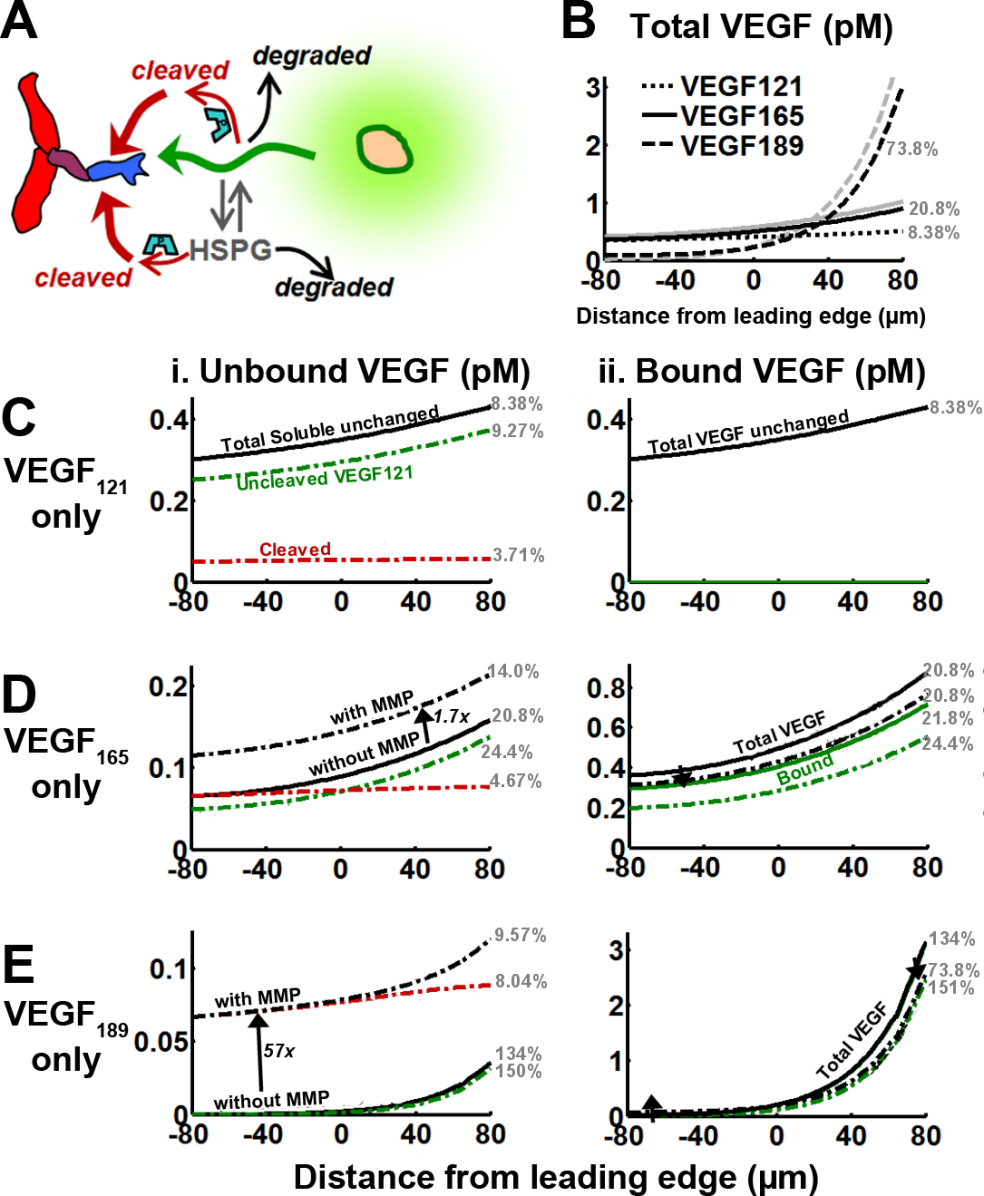
**Effect of VEGF-cleaving MMPs on the VEGF distribution**. Proteases (MMPs) were added to the matrix-sequestered VEGF degradation system at k_P _= 2.8·10^-4 ^s^-1 ^for systems expressing VEGF_121 _(C), VEGF_165 _(D), or VEGF_189 _(E). We look at the components of the soluble VEGF distribution (i), and bound and total VEGF (ii), both before the addition of proteases (solid lines), and after (dashed lines). The total VEGF distributions of different isoforms, in the presence of proteases (B, black lines), are compared to those in the absence of protease (B, grey lines). Simulations were performed in the absence of receptors (parameters given in Table 4). Isoform fractional gradient values at the tip cell are noted in grey with units %/40 μm.

VEGF cleavage (generating sizeable fragments) is also an important modulator of vascular patterning and experimentally, increased cleavage seems to mimic the effects of reduced HSPG binding affinity: increase in soluble VEGF, increase in range of the VEGF signal with a simultaneous decrease in the magnitude of the VEGF gradient, decrease in matrix-bound VEGF, and possibly, preservation of the total tissue VEGF levels [7,12,13,17,19,21]. Here we consider the role of VEGF-cleaving proteases on the VEGF distribution of the different isoforms for two different scenarios: the HSPG-binding-only model, which provides a reference case (Figure 5) and the model including matrix-sequestered VEGF degradation (Figure 6), which we saw above can result in VEGF isoform gradients similar to those observed experimentally. We assume VEGF cleavage can act on both soluble and matrix-bound VEGF.

Surprisingly, inclusion of proteolytic cleavage did not affect total soluble VEGF levels in the HSPG-binding-only model, despite reducing levels of uncleaved (full-length) VEGF and producing VEGF_114_, a non-HSPG binding diffusible isoform (Figure 5Ci,Di,Ei,). Mathematical analysis indicates that this effect is similar to the HSPG binding independence noted earlier. Proteases are predicted to reduce matrix-bound VEGF levels (Figure 5Dii,Eii), due to a proportional decrease in uncleaved VEGF, which is accompanied by a significant steepening of these distributions (fractional gradients are noted next to the lines on Figure 5). Comparing the different VEGF isoforms, proteases cleave VEGF_189 _much more thoroughly than VEGF_165_, while total VEGF in the VEGF_121 _system (Figure 5Cii) is not affected by proteolysis because its cleavage produces an equivalent isoform. This potentiation of VEGF proteolytic cleavage by HSPG binding is due to increased residence time of the matrix-binding isoforms.

In contrast, with the inclusion of matrix-sequestered VEGF degradation (Figure 6), proteolytic release of VEGF resulted in *increased *soluble VEGF levels, size of this increase was proportional to the matrix affinity of the uncleaved isoform (Figure 6Ci,Di,Ei). Furthermore, both the soluble VEGF and total VEGF distributions have reduced gradients (Figure 5Hi,ii) indicating that VEGF gradients can be disrupted by proteases in this model. Matrix-bound VEGF decreased due to the action of protease; however, its decrease was not as significant as in the HSPG-binding-only model, due to the existence of VEGF degradation processes. Overall, the total VEGF amount in each tissue is well preserved by the action of proteases (in the presence of proteases, VEGF_121 _system: 229 molecules; VEGF_165 _system: 380 molecules; VEGF_189 _system: 428 molecules; compare to Figure 4D levels; refer to Figure S3.1).

We note that in each case, cleaved VEGF has a nearly flat concentration profile, a consequence of it not being secreted at a single location. This contrasts with the uncleaved soluble and bound fractions, which are proportional to each other and are sharpened by proteases. Another surprising finding is that in the presence of isoform-specific degradation, the concentration of cleaved VEGF is similar in each isoform system (Figure 6Ci,Di,Ei). As described in more detail in Additional file 1, *section S2*, this is a result of total VEGF being preserved, indicating that the residence time of each isoform, and hence the time available for cleavage to occur, is similar.

### Correlating VEGF patterning to vascular patterning suggests total soluble VEGF may be an optimal VEGF signal

**Figure 7:**
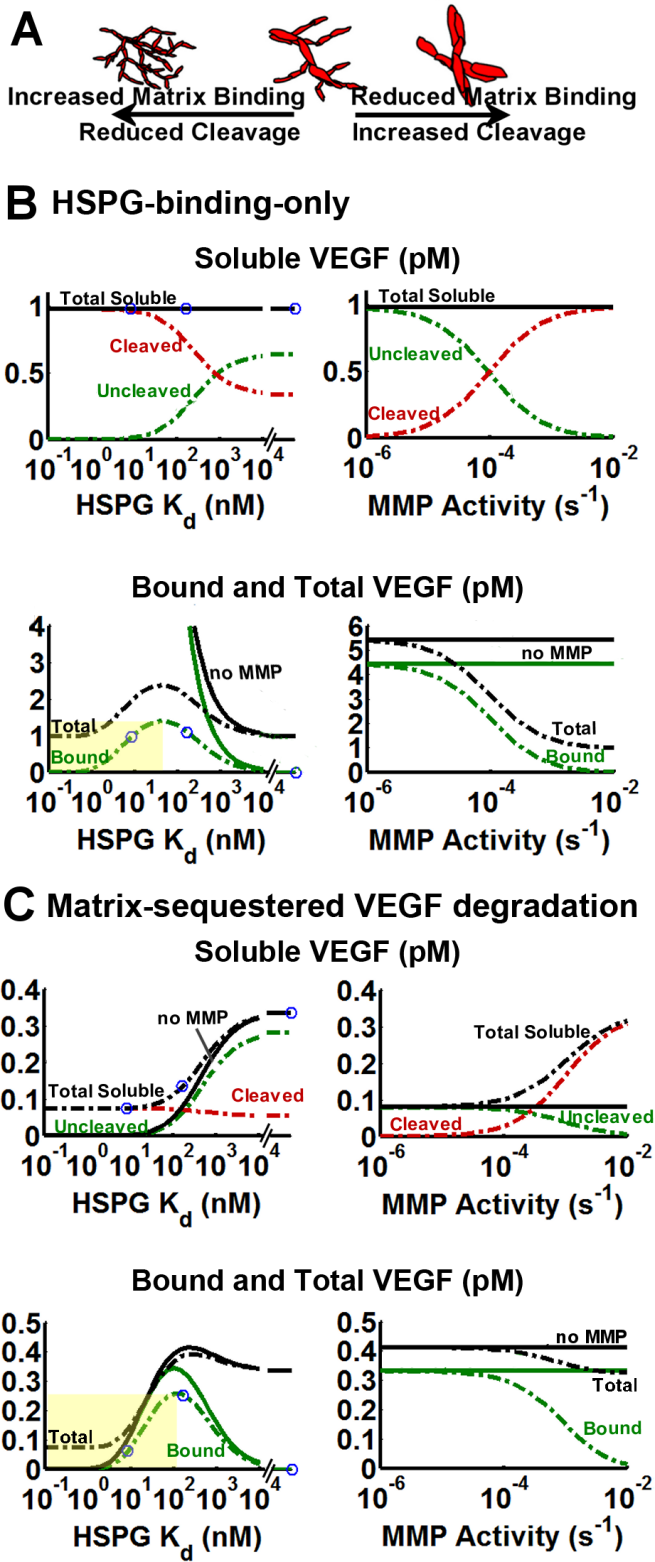
**Competing influences of HSPG binding and VEGF cleavage on biological responses**. Experimental evidence indicates that HSPG binding and VEGF cleavage modulate the same axis of vascular phenotypes (A) [6,7,13,17]. To understand how the VEGF distribution may be responsible for vessel patterning, we measured various metrics of the VEGF distribution as they would vary either with variation in the HSPG affinity of the secreted isoform (with MMP activity held constant at k_P _= 2.8·10^-4 ^s^-1^, dash-dotted lines) or with variation in the background proteolytic activity (k_P _ranged from 10^-6 ^s^-1 ^and 10^-2 ^s^-1^, assuming that VEGF_165 _is secreted). The HSPG-binding-only model (B) and the matrix-sequestered VEGF degradation model (C) were considered. Circle markers indicate respective positions of (from left to right) VEGF_189_, VEGF_165_, and VEGF_121 _(K_d _= ∞). Control cases simulated in the absence of MMPs are given in solid lines. Yellow boxes in B and C indicate the range where bound VEGF does not display behavior consistent with the experimental data. Simulations were performed in the absence of receptors, the presence of which would have a negligible impact on the VEGF distribution, to obtain computational efficiency permitting simulation of a broad range of parameters (parameters given in Table 4).

Using the VEGF patterning *in vivo *as recapitulated in the model, we now ask whether there is a viable VEGF-based guidance cue for cell sensing that is consistent with the twin observed properties: monotonic increase in vascular density with isoform length; and the opposite effect of VEGF cleavage (Figure 7A). We present results for VEGF near the tip cell (z = -40 to 0 μm), with an extended range of VEGF isoforms and MMP activity levels to compare, side-by-side, the isoform dependence and the MMP-based proteolytic dependence (Figure 7B,C).

In the HSPG-binding-only model (Figure 7B), total soluble VEGF is constant, and thus is unlikely to carry relevant information regarding vessel patterning. Instead, such information could come from changes in: soluble uncleaved VEGF; cleaved VEGF; or matrix-bound VEGF. In order for a signal to recapitulate the isoform and MMP dependence shown in Figure 1B, it should behave in the same fashion to decreasing HSPG affinity as it does to increasing MMP activity. In the context of Figure 7, that means that the behavior moving to the right along the x-axis should be the same on the HSPG and MMP graphs. Here however, levels of soluble uncleaved VEGF increase monotonically as the HSPG affinity of the secreted isoform decreases (due to decreased residence time in the ECM), but decrease as MMP activity increases. Cleaved VEGF (Figure 6B, red lines) is also inconsistent. Matrix-bound VEGF, however, has a biphasic behavior; at the tip cell, isoforms with intermediate and lower affinity to HSPGs satisfy the requirement that MMP effects oppose HSPG binding effects, but above an affinity limit this is violated (Figure 6B, yellow shaded region). This biphasic effect arises because greater matrix binding affinity results in greater accumulation close to the source of secretion, but also more rapid deterioration of the signal in space caused by proteolysis over a longer residence time. This effect was also seen in Figure 5B, as total VEGF_189 _levels fall below those of VEGF_165 _at the tip cell. Note that the closer the tip cell is to the source, the greater the likelihood that the monotonic behavior between isoforms is maintained, indicating that the distance from the VEGF source, all other factors being equal, may be a major determinant of isoform-specific vascular patterning.

In the matrix-sequestered VEGF degradation model (Figure 7C), soluble uncleaved VEGF, cleaved VEGF, and matrix-bound VEGF follow similar behavior as in the above HSPG-binding-only model. However, total soluble VEGF is not constant in this model. Interestingly, while neither soluble uncleaved nor cleaved VEGF are individually viable guidance cues, their sum (total soluble VEGF), displays the desired behavior, increasing with decreased isoform affinity to the matrix *and *with increased MMP activity, though at sufficiently high affinities, the differences at HSPG affinities larger than that of VEGF_165 _are not large. In addition, this behavior is present over the entire range of parameters, indicating that it is robust in its ability reproduce vascular patterning.

In both models (Figure 7B,C), total VEGF and total uncleaved VEGF (i.e. soluble uncleaved VEGF + matrix-bound VEGF) (*not shown*), display similar biphasic behaviors as does matrix-bound VEGF and thus do not enhance the signal provided by the matrix-bound form. The model also predicts the magnitude of VEGF gradients present near the tip cell. While fractional gradients appear to be significantly poorer candidates for guidance cues than are VEGF concentrations, absolute gradients seem to be as effective as VEGF concentrations (Additional file 1, *section S3; Figs. S3.2*, *S3.3*). Absolute gradients greatly extend the range of isoforms for which matrix-bound VEGF in the HSPG-binding-only model displays isoform monotonicity (Additional file 1, *Figure S3.2Biii*), however in the matrix-sequestered VEGF degradation model, it seems to show little variation with respect to changes in MMP activity, indicating weak HSPG/MMP antagonism (Additional file 1, *Figure S3.3*).

### Effects of receptor binding on isoform monotonicity and HSPG/MMP antagonism

Endothelial cells respond not to VEGF gradients directly but to VEGF receptor activation. Can VEGF receptors accurately sense total soluble VEGF, which we identified as a potential cue, or do other extracellular signals become relevant? Note that in the absence of NRP1 expression, VEGFR2 and VEGFR1 binding will be similar to the soluble distributions given in Figure 7, because uncleaved and cleaved VEGF isoforms bind similarly to the VEGF receptors. The presence of NRP1 may compensate for the inability of longer isoforms to diffuse far in tissues.

**Figure 8:**
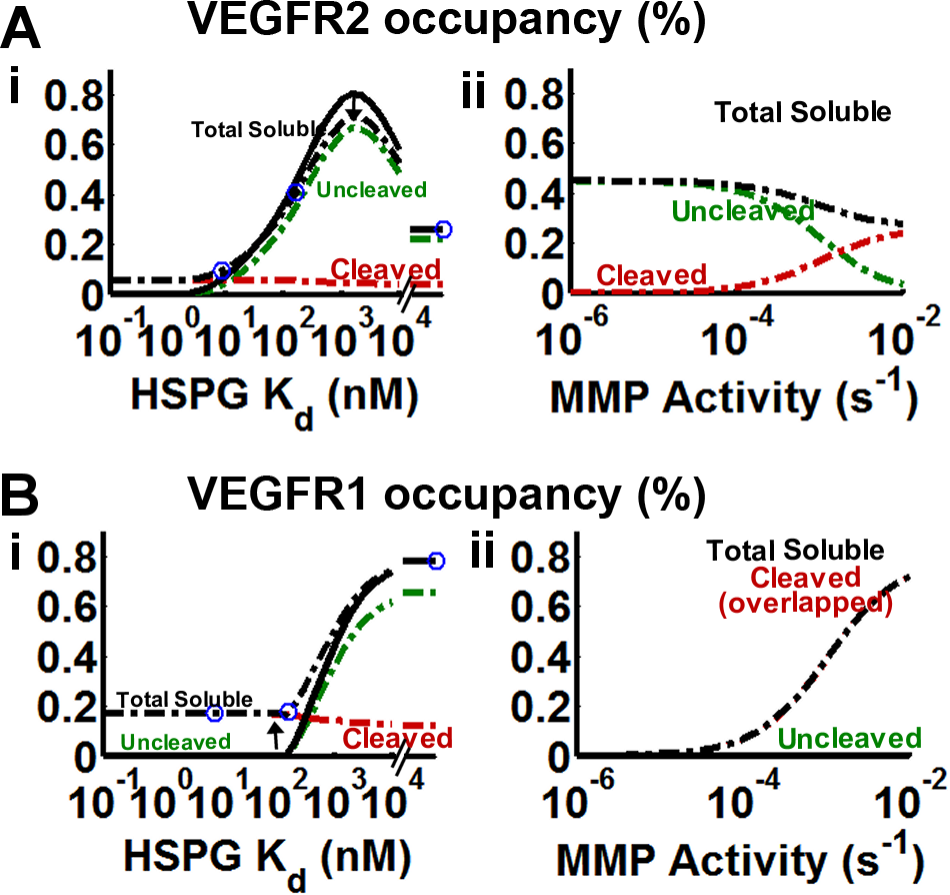
**VEGFR2 and VEGFR1 signaling in mediating vascular phenotypes**. Using the VEGF distributions calculated by the isoform-specific degradation model in Figure 7 we calculated VEGF binding to VEGFR2 (A) and VEGFR1 (B). We assume 10^4 ^VEGFR2, 10^4 ^VEGFR1, and 3*10^4 ^NRP1 per tip cell (assuming no VEGF depletion by the sprout). VEGF isoforms show isoform-specific differences in their ability to bind NRP1 and the VEGFR1-NRP1 complex. To calculate the receptor binding parameters for arbitrary isoforms, we imposed two constraints to interpolate/extrapolate around the known VEGF isoforms (refer to *Methods, Kinetic Parameters*). VEGF/NRP1 affinity was made to recapitulate the lack of binding of VEGF_121 _and stronger binding affinity of VEGF_189 _[37]. VEGF binding affinity to the VEGFR1-NRP1 complex and NRP1 coupling to the VEGF-VEGFR1 complex was set such that VEGF_121 _can fully bind to VEGFR1-NRP1 [95], while any isoform with greater HSPG affinity than that of VEGF_165 _cannot bind. Circle markers indicate (from left to right) VEGF_189_, VEGF_165_, and VEGF_121 _(K_d _= ∞).

We imposed the VEGF distributions calculated in Figure 7 onto our model of VEGF receptor interactions (refer to Additional file 1, *section S4.1*). VEGFR2 binding was similar for the matrix-sequestered VEGF degradation model (Figure 8) and the HSPG-binding-only model (*not shown*). VEGFR2 binding shows greatest binding at isoform affinities between VEGF_121 _and VEGF_165_, violating isoform monotonicity (Figure 8Ai), a result of the isoform-specific potentiation of the underlying uncleaved VEGF by NRP1. At high enough matrix affinities this is, however, unable to compensate for the low levels of uncleaved VEGF seen for these isoforms. We note that uncleaved VEGF_189 _(and possibly other long isoforms) cannot bind VEGFR2 [46], though its cleavage product can; this does not significantly alter the shape of the curve (Figure 8Ai), as most VEGF_189 _is cleaved (Figure 7C)

We note that when the uncleaved isoform exhibits strong HSPG binding, total soluble VEGF is due primarily to cleaved VEGF, and when the uncleaved isoform has minimal ECM binding, then cleaved VEGF is not significant (Figure 7C). A similar feature was noted in VEGFR1 binding in the presence of NRP1 (Figure S4.1A). As a result, we also tested the potential role of VEGFR1 signaling in recapitulating total soluble VEGF signaling (Figure 8B). Total VEGFR1 binding displays a greater range of isoform monotonic behavior than VEGFR2 binding, however, it is limited only up to the VEGF_165 _isoform, beyond which VEGFR1 binding remains flat or may actually increase with increased matrix-binding affinity (*refer to *Additional file 1, *Figure S3.3Dii*).

## Discussion

**Figure 9:**
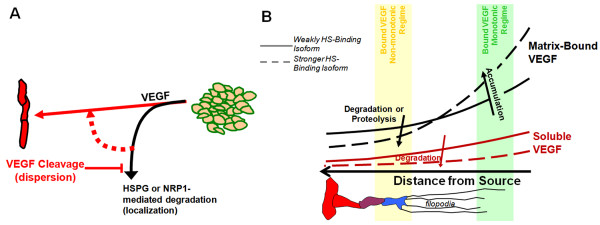
**Summary of VEGF transport results**. **A**, We propose that isoform-specific patterning in tissues results from differences in their rates of degradation (i.e. isoform-specific degradation), potentially mediated by HSPG or NRP1 binding. For longer isoforms to have more localized VEGF distributions [13], their degradation must be more rapid. We hypothesize that proteases enhance the spatial propagation of VEGF (i.e. VEGF redistribution) [16,17,19] by inhibiting this isoform-specific VEGF degradation. **B**, Role of matrix-binding affinity on VEGF patterning. Our computational model shows that HSPG-binding affinity affects soluble and matrix-bound VEGF in different manners. Increased matrix binding affinity universally decreases total soluble VEGF, in the case of HSPG-mediated isoform-specific degradation. However, matrix-bound VEGF shows increased "localization", a result of two competing behaviors: an increase in concentration due to accumulation close to the source of secretion, and a decrease in concentration farther away due to an increased rate of degradation. This causes matrix-bound VEGF to lose its isoform-monotonic behavior. The ordering of how isoforms are perceived is dependent upon the distance that sensing occurs from the VEGF source.

Our study proposes an important reinterpretation of the role of HSPGs and VEGF-cleaving proteases in the control of VEGF patterning *in vivo*. Our central proposition is that differential isoform degradation (or clearance), and not a difference in diffusion arising from HSPG binding, controls the spatial localization of VEGF in tissues (Figure 9A).

In the developing hindbrain system, VEGF_120 _distributes in a much more disperse manner (Figure 1A) than VEGF_164 _[13]. We find that while VEGF_164 _may diffuse slower in tissues than VEGF_120_, differences in grading will only arise when VEGF_164 _is also degraded more rapidly by the surrounding hindbrain parenchyma, possibly due to specific uptake by cell surface HSPGs or NRP1, preventing it from diffusing far (Figure 9A). We propose that this difference in degradation rates between isoforms (which we term isoform-specific degradation, and is here a result of degradation of matrix-bound VEGF) also explains why secretion of non-heparin binding isoforms such as VEGF_113 _or VEGF_120 _leads to greater VEGF levels in the solution phase of tissues than secretion of heavier isoforms, given identical secretion rates [7,21]. Note that assuming a simpler mechanism of reversible HSPG binding in an *in vivo *system is not able to explain this behavior at steady state (Figure 4A). Importantly, our mechanism of isoform-specific degradation also provides a general explanation for the phenomena of protease-mediated redistribution of VEGF activity: proteases, by either cleaving VEGF [7], cleaving HSPGs [15], or by cleaving VEGF inhibitors [32], inhibit the degradation of VEGF (Figure 9A), thereby leading to the accumulation of VEGF in tissues and an increased range of receptor activation on the vasculature. This hypothesis is distinct from what is commonly noted in the literature as VEGF release, in which VEGF is focally released from the matrix by the action of proteases and diffuses to activate vasculature.

The second thrust of our current work is the elucidation of how VEGF isoforms and VEGF-releasing proteases might regulate vascular morphology (Figure 1B). We originally asked the question: which VEGF metrics may be responsible for guiding vascular patterning, to recapitulate both isoform monotonicity [6,12,13,25] and the protease dependence in vascular patterning [6,7]? We identified several possibilities including the levels of matrix-bound VEGF, soluble VEGF levels, NRP1 dependent signaling, and even VEGF gradient directionality. Using the results of our model, we can now provide a more specific answer to this question.

For example, the model predicts as expected that VEGF_188_-secreting tumors would have the greatest levels of peritumoral matrix-bound VEGF [6], however, farther away at the vascular front, matrix-bound VEGF levels may actually be lower than if VEGF_165 _were being secreted (Figure 9B). Thus, how a sprout experiences the ordering of the isoforms by sensing matrix-bound VEGF may not always be monotonic with respect to the heparin binding affinity of the VEGF isoform; it will be dependent on the distance between the sprout and the VEGF source (Figure 9B). (Note that the specific ordering depends on HSPG concentrations, protease levels, degradation rates, etc. Given their intrinsic variability in biological systems, the conclusion that a sufficiently strong matrix-binding isoform will have less matrix-bound VEGF is robust.) Instead, the possibility that VEGF_189_-secreting systems also have the lowest levels of soluble VEGF of the different isoform-expressing systems seems to be consistent, even in the presence of proteases (Figure 9B).

An important alternative to the concentration of matrix-bound VEGF levels may however be its directionality, especially as VEGF_189 _seems to have the sharpest distributions in the absence of proteases (Figure 4Diii). While this holds true in the absence of proteases and can possibly explain observations in the mouse hindbrain [13], this metric does not account for the effect of VEGF-cleaving proteases, which further sharpen the distribution, instead of behaving in a HSPG-antagonistic fashion (*Figure S4.3A*). VEGF_189 _also may have stronger NRP1 binding than VEGF_165 _[37], however our results show that soluble uncleaved VEGF_189 _levels may be so low (the majority will be cleaved or degraded), that total NRP1-potentiated VEGFR2 signaling may be weaker than that in VEGF_165_-secreting systems. In fact, our model shows a surprising result: VEGFR1 binding of total soluble VEGF seems to better reconcile both isoform monotonicity and the antagonistic relationship between HSPG affinity and MMP activity than VEGFR2 binding does (Figure 9).

Our results may provide insight into the nature of several experimental observations regarding vascular patterning. A comparison of our results to those of Ruhrberg et al's hindbrain data suggest that while the overall VEGF distribution is spatially non-monotonic (Figure 1A) [13], the underlying soluble fraction of VEGF is monotonic and is the basis for endothelial behavior. We note, however, that several important biological effects need to first be taken into account, for example the roles of filopodia [12,13,74] and the direct receptor signaling of matrix-bound VEGF [35].

The loss of monotonicity for matrix-bound VEGF (Figure 7B, Figure 9B) is a result of the isoform-dependent decrease of the soluble uncleaved VEGF fraction in space. We now discuss how the tip cell filopodia can contribute to the process of isoform sensing. By projecting out in front of the tip cell, filopodia may be able to detect a region of space where matrix-bound VEGF may in fact operate in an isoform-monotonic manner (Figure 9B), effectively increasing the spatial range of the matrix-bound VEGF fraction's isoform monotonicity. We conceptualize this to sprouting angiogenesis. In the initial stages of sprouting where the sprout is far away from the VEGF source, only soluble VEGF will exhibit isoform monotonicity. However, as a sprout continues to invade closer into the VEGF secreting tissue, matrix-bound VEGF signaling and VEGF gradients will also exhibit the correct isoform monotonicity (refer to Additional file 1, *section S3.2, Figure S4.4*). These effects would be relevant *in vivo *as VEGF gradients never seem to be more than 50 μm in front of the vascular front [12,13], a range that can easily be sensed by filopodia [12]. Thus, while total soluble VEGF is theoretically the most isoform-monotonic signal, there may be no practical differences between it and matrix-bound VEGF. In fact, the information provided in matrix-bound VEGF may be even more relevant than that of soluble VEGF as it can result in differential VEGFR2 signaling that favors activation of p38/MAPK compared to soluble VEGF [35] and may mediate increased interaction with cell-surface NRP1 [34], both of which may play a direct role in branching and migration behaviors leading to increased vascular density. Unlike the behavior of matrix-bound VEGF, an isoform monotonic signal in NRP1-mediated VEGFR2 signaling does not necessarily emerge as a sprout invades into the VEGF secreting tissue (a result of reaction limitations in VEGF/VEGFR2/NRP1 coupling) (*Figure S4.4C*), indicating that NRP1-mediated VEGFR2 signaling may be less capable of giving rise to isoform-specific increases in vascular branching complexity. However, this conclusion is based on assuming a model where receptors only bind soluble VEGF; how the signaling of matrix-bound VEGF changes these conclusions is not known.

Our results indicated that VEGFR1 signaling of soluble VEGF may be able to also mediate isoform-specific differences in vascular patterning. However, this conclusion is at odds with the VEGFR2-dependent nature of angiogenesis in the retina [12], in *in vitro *patterning of porcine aortic endothelial cells [7], and in MMP9 induced carcinogenesis [16]. In addition, VEGFR1 signaling supports migratory behavior through p38/MAPK [26], however our results suggest an inhibitory role of VEGFR1 signaling on vascular density (Figure 8Bi). While our model cannot elucidate the importance of VEGFR1 signaling, it is interesting to note that when VEGFR2 signaling is monotonically increasing with the isoform length, VEGFR1 signaling is decreasing, suggesting that a balance between the two receptors may be important.

The unique role of matrix-bound VEGF in mediating the branching phenotype through filopodia may offer an interesting solution to the paradoxes of VEGF-cleaving MMPs in tumor growth. For example, both VEGF_188 _tumors and VEGF_164Δ108-118 _tumors are similar in that they have higher intratumoral vascular density and lower-diameter vessels compared to VEGF_164 _tumors; however, whereas VEGF_164Δ108-118 _tumors are markedly more proliferative than VEGF_164 _tumors [7], VEGF_188 _tumors show negligible growth [6]. We may conclude that this discrepancy indicates that the resulting vascular beds in the two tumors are different in ways that the intratumoral vessel density cannot measure, for example, in their connectivity to peritumoral vasculature [6], which may be a limiting factor for nutrient delivery; we propose that a separate aspect of VEGF signaling controls this latter behavior. Our model suggests a crucial difference between the two tumors: while VEGF_188 _tumors show minimal VEGFR2 activation, even less than that of VEGF_120 _tumors, VEGF_164Δ108-118 _tumors instead show even higher VEGFR2 activation than VEGF_164 _tumors (Figure 8A). As a result, it may be possible that the ability of the vasculature to support tissue growth may be dictated by VEGFR2 activation at the vessel itself, due to the receptor binding behavior of soluble VEGF, as suggested in numerous studies [16,17]. Furthermore, notice that peak VEGFR2 activation occurs between the VEGF_165 _and VEGF_121 _isoforms (Figure 8Ai). This may support the observation that VEGF_120 _is at least as tumorigenic as VEGF_164 _in some systems due to intrinsic differences between systems [25,39]. Overall, we hypothesize that intratumoral vessel density and branching is determined by matrix-bound VEGF detected by filopodia removed from the vessel surface, while vessel efficacy is dictated by VEGFR2 activation at the vessel body. As a result, we thus posit that the tumorigenic behavior ultimately depends on the ability of VEGF to keep its receptor and NRP1 binding domains intact. The inability of VEGF_188 _tumors to elicit VEGFR2 activation, despite it potentially having higher affinity to NRP1 than VEGF_164_, is a result of its degradation and cleavage (Figure 7C).

This hypothesis may also explain the paradoxical roles of specific proteases in tumorigenesis. Our mechanism of MMP-mediated VEGF redistribution (Figure 9A) shows that proteases will increase the functional soluble VEGF concentration by inhibiting the process of VEGF degradation. However, while several modes of VEGF redistribution, such as through MMP9, heparinases, or VEGF inhibitor cleavage are implicated as pro-tumorigenic, mediating an angiogenic switch [15,16,19,20,29,30,32], there are several notable exceptions where protease activity instead leads to diminished tumor growth [7], an effect similar to the plasmin-mediated loss of wound healing due to a loss of angiogenesis [75]. We note an important trend: in studies where pro-tumorigenic behavior occurs from VEGF release, VEGF has not been shown to be directly cleaved. Bergers et al have shown that MMP9 mediates VEGF-release induced carcinogenesis in pancreatic islets [16] without determining the mechanism of release. However, subsequent studies have shown that MMP9 does not necessarily cleave VEGF [15,31-33], as suggested by [7], but instead acts to cleave HSPGs directly [15]. In fact, Joyce et al [30] show that MMPs and heparinases have similar effects in the pancreatic islet system, which strengthens the argument that the MMP9 induced angiogenic switch in [16] may have been mediated by HSPG cleavage. In contrast, studies where proteases reduce the angiogenic potential of VEGF show direct evidence of VEGF being cleaved and/or degraded [7,75,76]. We propose that VEGF needs to maintain coreceptor domains for effective tumorigenesis. Cleavage of VEGF, while increasing the total soluble VEGF concentration, may decrease overall VEGFR2 activation due to a loss of NRP1 binding (recapitulated in Figure 8Aii); heparinases, MMP9, and VEGF inhibitor proteases also prevent VEGF degradation but redistribute intact, coreceptor-binding VEGF.

Our results suggest that a central facet of VEGF patterning *in vivo *is its degradation, which we show necessarily occurs in an isoform-specific manner. Several mechanisms may underlie VEGF's isoform-specific degradation, and it is not currently known what, if any, mechanisms operate in the different *in vivo *experimental systems used. Interstitial cells and endothelial cells from nearby vessels may uptake VEGF isoform in an HSPG- or NRP1- dependent manner. This is supported by observations that NRP1 and VEGF receptors are typically present in many types of parenchyma, e.g. hindbrain [77], astrocytes [78], tumor [27,37], and skeletal muscle [79]. On the other hand, degradation may be due to the action of VEGF-degrading proteases, possibly the same proteases that also initially cleave VEGF; however, this possibility does not seem to be consistent with developmental systems where VEGF cleavage has not been detected [17]. An interesting possibility that has recently been raised is that of VEGF inhibition by soluble VEGF inhibitors, e.g. sVEGFR1 [11,33] or connective tissue growth factor [32], operating in an isoform-dependent manner through either HSPG complexation or through NRP1 complexation. Interestingly, each of these mechanisms also supports the ability of proteases to allow VEGF to escape degradation, through either HSPG binding or NRP1 binding, mediating VEGF redistribution. An important uncertainty in VEGF catabolism is whether endothelial cells represent the primary source of VEGF receptors and degradation *in vivo *or not. For example, while the background neural progenitor cells in the hindbrain may express NRP1 [78], endothelial expression is typically thought to be very strong [80]. Furthermore, immunochemical staining usually shows that VEGF concentrations diminish precisely at the vascular front [12,13].

Besides being able to reproduce experimental observations of VEGF patterning *in vivo*, is there evidence for isoform-specific degradation in tissues? VEGF (specifically, VEGF bioactivity) has been shown to degrade *in vitro *under cell culture conditions [53,81] and in fact, proteases that cleave VEGF into shorter isoforms also seem to degrade it further [51,82]. In addition, while VEGF degradation has not typically been studied as a cause of VEGF patterning, this view is commonly accepted in numerous developmental systems (e.g. Decapentaplegic, Wingless in Drosophilia) [52,83,84]. Evidence for isoform-specific degradation however may come from an indirect source. Perlecan knockdown in zebrafish establishes a diffusible VEGF phenotype [85]. Surprisingly, it also increased total tissue VEGF levels. Relevant to the present study is the fact that total VEGF levels did not decrease, supporting the view that HSPGs may be important mediators of VEGF degradation. However, there are other pieces of evidence that seem to contradict isoform-specific degradation: intravenously-injected bevacizumab shows significantly greater tumoral deposition in VEGF_189_-expressing tumors compared to VEGF_165 _tumors and VEGF_121 _tumors [86]. Preliminary computational results suggest that this observation supports the HSPG-binding-only model on the basis of the total number of bevacizumab binding sites, i.e. VEGF, increases proportionally to the VEGF isoform matrix affinity.

An interesting prediction of the isoform-specific degradation model is that the extracellular residence times of different VEGF isoforms are roughly equal. Thus, a test of the model can be made by injecting labeled VEGF isoforms into tissues and measuring their half-lives in the interstitial fluid or lymph. If this test shows that the longer isoforms have significantly greater residence times in tissues, it would disprove the isoform-specific degradation model. The residence time of VEGF in tissues is a direct measure of the overall degradation and clearance rate, and its constancy is identical to the statement that the total levels of VEGF in tissue are roughly constant against differences in patterns of isoform secretion and VEGF proteolysis, a finding that is suggested by results in [7,19]. Note that this statement, however, does not contradict isoform-specific degradation. In this model, the rate of degradation (or clearance) of the soluble fraction of VEGF is isoform specific, with heavier isoforms showing more rapid degradation; however, accounting for all phases of VEGF (e.g. matrix bound, receptor bound), the average rate of degradation of any VEGF isoform is nearly identical (refer to Additional file 1, *section S2*).

Overall, our results form the basis for a different view of VEGF patterning and endothelial behavior in response to VEGF. The assumption behind how VEGF patterning is intuitively interpreted is that of the transient: transiently, MMPs elicit VEGF release, which can increase VEGF receptor signaling on endothelial cells [16], and HSPGs do hinder diffusion, forming isoform-specific differences in soluble VEGF patterning. However, this assumption ignores what happens to the VEGF distribution over much longer periods of time, which are likely just as important for slowly evolving processes such as vascular patterning. The transient assumption only seems valid in studying *in vitro *systems, systems where VEGF is cleared very slowly [66]. On the other hand, *in vivo *systems seem to represent a major phenomenological difference due to their much more rapid VEGF dynamics (τ < 1 h, Appendix A1), necessitating a steady-state analysis. Several additional assumptions have also been made in our analysis (Table 1). For example, we assumed that the intrinsic proteolytic cleavage rate of all isoforms is identical. However, experimental studies indicate that VEGF_189 _may be more resistant to MMPs than VEGF_165 _[7]. This point is interesting to note since another study, [51], found that VEGF_111 _(a form of VEGF also resistant to degradation or cleavage) also results in angiogenesis with high vascular density [51], similar to VEGF_164Δ108-118 _formed vessels [7]. Since the inability to be further cleaved/degraded may be the common theme, it may indicate that an inability to be cleaved/degraded alternately underlies higher vascular densities in those systems. Finally, in the current study, we specifically tested HSPG binding as the mechanism of isoform specificity in VEGF degradation. However, our results do not change if the isoform-specific degradation occurs solely through soluble VEGF being degraded in an isoform-specific manner (*not shown*), such as in a NRP1-dependent fashion.

## Conclusions

In the present study, we have identified a general mechanism of VEGF transport that explains experimental data regarding VEGF isoform patterning and proteolytic release at steady state, that of isoform specific degradation. We have further highlighted possible mechanisms by which information in the VEGF distribution can be used to guide vascular patterning in an attempt to explain vascular branching complexity and also the regulation of angiogenesis and tumor growth by proteases. A major limitation of our present study is that we were only able to broadly consider isoform ordering with respect to one or two features of the VEGF distribution. Instead, sprout formation and the subsequent patterning may be the result of a complex, temporal orchestration of multiple extracellular VEGF fractions, receptor signaling states, and cell types. Such an analysis is outside the scope of our study, however rule-based cell models and other modeling efforts are being developed to the sophistication required to address the multifactorial problem of vascular patterning, e.g. [87-89]. Many studies implement VEGF degradation and/or ECM binding and proteolysis [90-93] however not in a form, that as we show, gives rise to differential VEGF gradients seen in vivo [13]. We believe that the advances made in our study, specifically the necessity of a mechanism of isoform-specific degradation, would be useful to such studies to improve the predictions of the VEGF distribution.

Important biological questions remain to be addressed, especially regarding the existence and nature of isoform-specific VEGF degradation, the nature of the MMP induced angiogenic switch, and the possible role of soluble inhibitors such as sVEGFR1 in sprout formation, isoform patterning, and in the protease-mediated angiogenic switch.

## List of Abbreviations

(VEGF, e.g. VEGF_121 _indicates isoform of length 121 amino acids): Vascular endothelial growth factor A; (ECM): extracellular matrix; (HSPG): heparan sulfate proteoglycan; (MMP): matrix-metalloproteinase; (VEGFR): VEGF receptor; (NRP): neuropilin; (FO): fractional occupancy; (AG): absolute gradient; (FG): fractional gradient; (IFG): isoform fractional gradient.

## Authors' contributions

PV carried out the calculations, performed the simulations and wrote the first version of the manuscript. All authors participated in the design of the study and the model formulation, analysis of the results and writing and editing the manuscript. All authors read and approved the final manuscript.

## Supplementary Material

Additional file 1 **Supplementary Methods and Results**. The supplemental material is used to provide parameter estimation of VEGF/HSPG binding affinities, provide in-depth methodology for the simulations, and derive important theoretical results. (File is in PDF format, readable with Adobe Reader). Click here for file
