# Supplementary material for: Formation of VEGF isoform-specific spatial distributions governing angiogenesis: computational analysis
Source: BMC Syst Biol. 2011 May 2;5:59. doi: 10.1186/1752-0509-5-59 (PMC3113235; doi:10.1186/1752-0509-5-59)
Supplement: Additional file 1 — Supplementary Methods and Results. The supplemental material is used to provide parameter estimation of VEGF/HSPG binding affinities, provide in-depth methodology for the simulations, and derive important theoretical results. (File is in PDF format, readable with Adobe Reader). [file 1752-0509-5-59-S1.PDF]

Supplemental Methods and Results

This supplemental material details the parameter estimation of VEGF/HSPG binding affinities, provides in-depth methodology for the simulations, and derives important theoretical results. It is organized into the following sections:

Section S1. Estimating the VEGF/HSPG binding affinity

S1.1 Heparin competition study of cell-secreted matrices in vitro (Houck et al.)

S1.2 Estimation of $VEGF_{189}$ binding affinity to heparin

Section S2. Detecting VEGF gradients with VEGF receptors

S2.1 Sensitivity of detecting a single VEGF isoform with VEGFR2

S2.2 Sensitivity in detecting two VEGF isoforms with a single receptor is dependent on the VEGF gradient

S2.3 Relationship between individual isoform sensitivities and overall gradients

S2.4 Two mechanisms reduce the VEGFR2 sensitivity in detecting $VEGF_{165}$ fractional gradients in the presence of Neuropilin-1

Section S3. Effect of clearance, degradation, and proteolysis on interstitial VEGF levels – compartmental analysis

Section S4. Extended results for isoform-specific degradation model

Section S1 – Estimating the VEGF/HSPG binding affinity

Exons 6 and 7 confer VEGF with basic residues that allow it to bind the heavily sulfated GAG chains of HSPG and heparin. $VEGF_{165}$ has been previously reported to bind to heparin with an affinity of $K_d \sim 165$ nM [1, 2]. Using our estimate of ~750 nM ECM binding sites (see Table 5 of main manuscript), these parameters are in agreement with the observation that approximately 80% of $VEGF_{165}$ is bound [3] (see *S1.1 Heparin competition study* below). In contrast, for $VEGF_{189}$, Houck et al. show virtually all of the isoform is bound to the ECM [3]. Two other studies reported $VEGF_{189}$ binding to be less strong: $VEGF_{165}$ – 67% free/total, $VEGF_{189}$ – 20% [4]; $VEGF_{165}$ – 67%, $VEGF_{189}$ – 33% [5]. However, these data were obtained from tumors grown *in vivo*, and the difference in apparent affinity may be due to the presence of endogenous proteases releasing a significant portion of ECM-sequestered VEGF. In the sections below, we attempt to estimate the $VEGF_{189}$ binding affinity to the HSPGs. While most studies are performed against heparin, binding to heparin has been shown to be similar to binding to heparan sulfate for bFGF [2, 6] and $VEGF_{165}$ [1, 7] (see Table S1). Note, however, that at least one group [8] observes dissimilar binding to heparin and HS.

S1.1 Heparin competition study of cell-secreted matrices in vitro (Houck et al.)

We used data from heparin competition studies against cell lines expressing one of the three major VEGFA isoforms [3] (Fig. S1A). For all three isoforms, heparin above 0.1 mg/mL caused a plateau in the free VEGF levels, which we assume represented the total extracellular VEGF in each system. We calculated the total HSPG binding site concentration, $[H]_{Total}$, by fitting the experimental data for $VEGF_{165}$-expressing cells with a previously determined value of $K_d = 165$ nM [2] to be ~700 nM. This calculation also suggested that $K_d \sim 165$ nM is a reasonable aggregate affinity estimate as significantly different K_d or HSPG values did not match the experimental data. We then used $[H]_{Total}$ to fit the experimental data for $VEGF_{189}$. Parameter estimation was done using a False-Position algorithm [9]. These experimental data are not accurate enough to allow determination of the $VEGF_{189}$ binding affinity, as any value of $K_d < 50$ nM was not significantly different from the data points.

S1.2 Estimation of $VEGF_{189}$ binding affinity to heparin

To estimate the $VEGF_{189}$ binding affinity to heparin/HS, we also correlated heparin-sepharose elution data with K_d measurements for $VEGF_{165}$ [1] and extrapolated to known data for $VEGF_{189}$, a technique that was demonstrated to be accurate for bFGF [10] (Fig. S1B).

Wild-type $VEGF_{165}$ has been previously reported to have a heparin affinity of $K_d = 165$ nM [2], whereas the [NaCl] required for elution against HSeph has been estimated at 0.68 – 0.9 M [3, 11]. Krilleke et al. have recently measured the $VEGF_{164}$ elution and K_d for wild-type $VEGF_{164}$ and two substitution mutants (K26A and R13A/R14A) displayed in Table S1 and Fig. S1B [1]. Following the method of Thompson et al. [10], we used this data to fit an exponential function relating the [NaCl] required for elution and K_d (Fig. S1B). Compared to bFGF, $VEGF_{164}$ has a steeper dependence of K_d for [NaCl] (an exponential coefficient of -7.7 compared to -3.8). We note that the exponential coefficient for VEGF is greater than that of bFGF by a factor of ~2.0. This may correlate with the valency of the HSPG binding, where VEGF is a homodimer and both C-terminal domains bind simultaneously [12], bFGF can only bind monovalently.

Plouet et al. [13] reported $VEGF_{189}$ elution from HSeph at 1.2 M NaCl, which is within a range previously determined by Houck et al. [3]. From the relation for $VEGF_{165}$ determined

above, the binding affinity of native $VEGF_{189}$ towards heparin is calculated as 8.5 nM, 19-fold greater affinity than $VEGF_{165}$. Thus, we find that $VEGF_{189}$ has stronger affinity than bFGF (K_d = 24 nM) supporting evidence that $VEGF_{189}$ is able to compete well with bFGF for matrix binding [14], if it were not for the fact that most of the ECM binding sites are unsaturated ($[V]/K_d^{189,HS} \sim 1.2 \cdot 10^{-4}$).

We should note that the extrapolation of $VEGF_{165}$ data to $VEGF_{189}$ is useful only as a first estimate. This is because the peptides encoded by exon 7 (used in the correlation above) are significantly different than those encoded by exon 6a (for $VEGF_{189}$). For example, exon 7 (PCGPC-SERRK-HLFVQ-DPQTC-KCSCK-NTDSR-CKARQ-LELNE-RTCR) comprises ~25% basic resides whereas exon 6a (KSVRG-KGKGQ-KRKRK-KSRYK-SWSV) comprises ~50% basic residues. It should be noted that $VEGF_{145}$ (which has exon 6a but not exon 7) seems to bind to ECM in a heparin-independent fashion [15]. Whether this is also true for $VEGF_{189}$ is not known. Additionally, the presence of exon 6a may render $VEGF_{189}$ too dissimilar from $VEGF_{165}$ for the above method to be useful. For example, $VEGF_{189}$ cannot bind VEGFR2 indicating potentially severe structural differences from $VEGF_{165}$ [13]. Finally, the heparin elution may not always be a reliable indicator of heparin affinity [1]. Two examples from VEGF point to this. $VEGF_{145}$ (and uPA-cleaved $VEGF_{189}$) has a similar [NaCl] for heparin elution as $VEGF_{165}$, but as discussed above, the binding seems to be HS- and heparinase-independent [15]. For $VEGF_{165b}$, binding to HS is negligible [16] but there is a modest salt elution (0.34 M – Table S1) [7].

Despite this, several lines of evidence support the order of magnitude of our estimate. The above estimate from Houck et al. [3] (*Section S1.1*) yields K_d < 50 nM, while similar estimates from Tomii et al. indicates a value of K_d = 17 nM [4] (*not shown*). Similarly, for $VEGF_{145}$, we obtain K_d estimates of ~94 nM [16] and ~50 nM [17], which are, as expected, larger than the K_d for $VEGF_{189}$ (i.e. lower affinity).

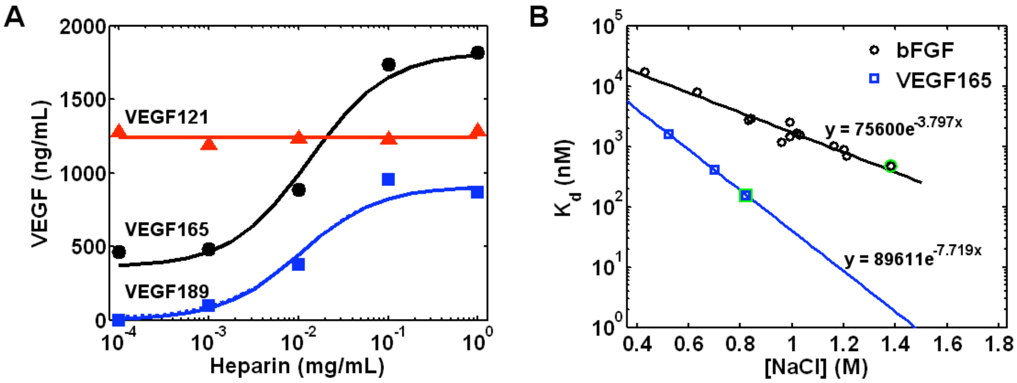

Figure S1.1. Estimation of $VEGF_{189}$ binding affinity to HSPG.
Experimental data from heparin-sepharose chromatography and heparin competition are used to estimate the binding affinity of $VEGF_{189}$ to HSPGs. Symbols: experiments; lines: simulations. **A**, In cell monolayer experiments, exogenous heparin competed VEGF isoforms off cell-associated matrix; released VEGF was then quantified using ELISA (markers) [3]. We used simulations to estimate total HSPGs available and the binding affinity of $VEGF_{189}$ (solid line: K_d = 8.5 nM; dotted line: K_d = 100 pM; note they are almost coincident). **B**, Experimental data for bFGF [10] and K_d-[NaCl] estimates for $VEGF_{165}$ [1]; lines show best fit to experimental data. The green markers indicate the binding affinity of the native species without modifications.

TABLE S1. Growth Factor Heparin/HSPG binding affinity				
**Ligand**	**Substrate**	**K_d (nM) at 0.15 M [NaCl]**	**Required [NaCl] (M) for Heparin/HS dissociation**	**Ref**
**$VEGF_{121}$ and cleaved VEGF**				
$VEGF_{121}$, $VEGF_{110}$	Heparin	N.S.	<0.15	[3, 11]
$VEGF_{165\text{-}110}$ heterodimer	Heparin	-	0.42	[11]
$VEGF_{165}$ C-terminal fragment	Heparin	-	0.69	[11]
$VEGF_{189}$ + uPA	Heparin	-	0.7	[13]
**$VEGF_{165}$ and substitution mutants**				
$VEGF_{164}$	Heparin	-	<0.9	[3]
$VEGF_{164}$	Heparin	165	-	[2]
$VEGF_{164}$	Glypican	0.12	-	[18]
$VEGF_{164}$	CD44-HSPG	333	-	[19]
$VEGF_{165}$	Heparin	91	-	[20]
$VEGF_{165}$	Heparin	-	0.68	[11]
$VEGF_{164}$	Heparin	157	EC_{50} = 0.82	[1]
$VEGF_{164}$, K26A	Heparin	415	EC_{50} = 0.70	[1]
$VEGF_{164}$, R13A/R14A	Heparin	1600	EC_{50} = 0.52	[1]
$VEGF_{165}$ immobilized	Heparin	1500	-	[7, 21]c
$VEGF_{165}$	Heparin	-	0.41, 0.65	[7]
$VEGF_{165b}$	Heparin	-	0.34	[7]
**Exon 6-containing VEGF**				
$VEGF_{145}$	HS	<94 nM	-	[16]
$VEGF_{145}$	Heparin	-	0.6-0.7	[15]
$VEGF_{189}$	Heparin	-	0.9 to 2.0	[3]
$VEGF_{189}$	Cell secreted matrix †	17 nM	-	[4]
$VEGF_{189}$	Heparin	-	1.2	[13]
FGF				
FGF-2	Heparin	23	-	[2]
FGF-2	Heparin	-	1.5 to 1.6	[22]
FGF-2	Heparin	460	1.38	[10]
FGF-2	Native ECM	-	3.0 – 5.0	[23, 24]
FGF-2	BM HSPG	23.6	-	[6]
FGF-2	CD44-HSPG	45	-	[19]
FGF-2	HSPG	7.5	-	[25]
FGF-2	Perlecan	70		[26]
FGF-7	Glypican	0.30	-	[18]

N.S.: not significant
- : not performed/provided
c : calculated from ref. using $K_d = [V]\cdot([H]^{Total}-[VH])/[VH])$
†: Matrix [HSPG] was calculated assuming $VEGF_{165}$ has K_d = 165 nM

Section S2 – Detecting VEGF gradients with VEGF receptors

Along with studying the formation of VEGF gradients in the extracellular milieu, it is also necessary to understand how effectively these gradients may be detected by VEGF receptors on the sprout surface. Specifically, we seek an understanding of the effects of receptor saturation, NRP1 expression, and multiple competing isoforms on the overall detection of VEGF gradients and sprout guidance. In our present model, we consider that only soluble VEGF can ligate VEGF receptors on the sprout surface. In the following sections, we label the uncleaved isoform as $VEGF_{165}$ and the cleaved isoform as $VEGF_{114}$, however the results hold for other combinations of isoforms.

S2.1 VEGF sensing by endothelial cells via VEGFRs and NRP1

We study the ability of VEGFR1 and VEGFR2 to sense VEGF gradients in the presence of NRP1. We impose a VEGF distribution of mean concentration 1 pM with a gradient of 5%/40 μm (this gradient prevents the artifact of VEGF depletion near the sprout). $VEGF_{114}$ and $VEGF_{165}$ are considered here; $VEGF_{121}$ binding should behave roughly similar to $VEGF_{114}$, because although it binds NRP1 [27], its affinity to NRP1 is significantly weaker than that of $VEGF_{165}$ and it cannot crosslink to VEGFR2 [27].

NRP1 potentiates $VEGF_{165}$ binding to VEGFR2 (Fig. S2.1Ai) and thus also increases the steepness of $VEGF_{165}$-VEGFR2 absolute gradients (Fig. S2.1Aii): for our baseline $3*10^4$ NRP1, 10^4 VEGFR2, 10^4 VEGFR1 per cell, our model predicts a difference of ~25 $VEGF_{165}$-VEGFR2 complexes over the length of the tip cell, versus only ~2.3 complexes difference in the absence of NRP1, or for $VEGF_{114}$. NRP1 diminishes $VEGF_{165}$ binding to VEGFR1, due to NRP1 coupling to VEGFR1, which prevents subsequent $VEGF_{165}$ binding to VEGFR1 [28], but has no effect on $VEGF_{114}$. In other words, VEGFR1 binding is stronger towards the non-heparin binding isoforms in the presence of NRP1. Despite increasing VEGF binding to VEGFR2, NRP1 decreases the fractional gradient of $VEGF_{165}$-bound VEGFR2 (Fig. S2.1Aiii) (i.e. a decrease in VEGFR2 sensitivity), an effect that is greatly more significant in the absence of VEGFR1 (*not shown*). The converse is true for $VEGF_{165}$-VEGFR1 fractional gradients.

Measuring the ability of VEGFR2 to detect a VEGF gradient is identical to measuring the VEGFR2 fractional occupancy (FO) at varying levels of VEGF (Fig. S2.1B); in fact, sensitivity can be defined as d ln(FO)/d ln([VEGF]) (*Appendix, Eqn. 28*). In the absence of NRP1, sensitivity reduces to $S_{FG\text{-}165} = 1 – FO_{165,VEGFR2}$ (Fig. S2.1Biii, red line). However, in the presence of NRP1, the expression is significantly more nonlinear (refer to *Section S2.5*). As seen in the contour map (Fig. S2.1Biv), sensitivity reduction occurs at two distinct receptor combinations: either at high VEGFR2 and intermediate NRP1 or at low VEGFR2 and high NRP1. Mathematical analysis indicates that the dual regions of sensitivity loss arise, respectively, from VEGFR2 saturation via NRP1 binding and NRP1 saturation via VEGFR2 binding, i.e. when forming the ternary complex (refer to *Section S2.5*). Interestingly, this shows that VEGFR2 sensitivity loss is not only a result of VEGFR2 saturation, but also an indirect result of NRP1 saturation, even when in the latter situation VEGFR2 is largely unoccupied. This is corroborated by eliminating individual reactions in the VEGF/VEGFR2/NRP1 pathway from the model (Fig. S2.1Bv), which removed the indicated sensitivity-loss regions in Fig. S2.1Biv. VEGFR1 reduces the intensity of NRP1-mediated sensitivity losses by competing away NRP1 from $VEGF_{165}$ binding (*not shown*).

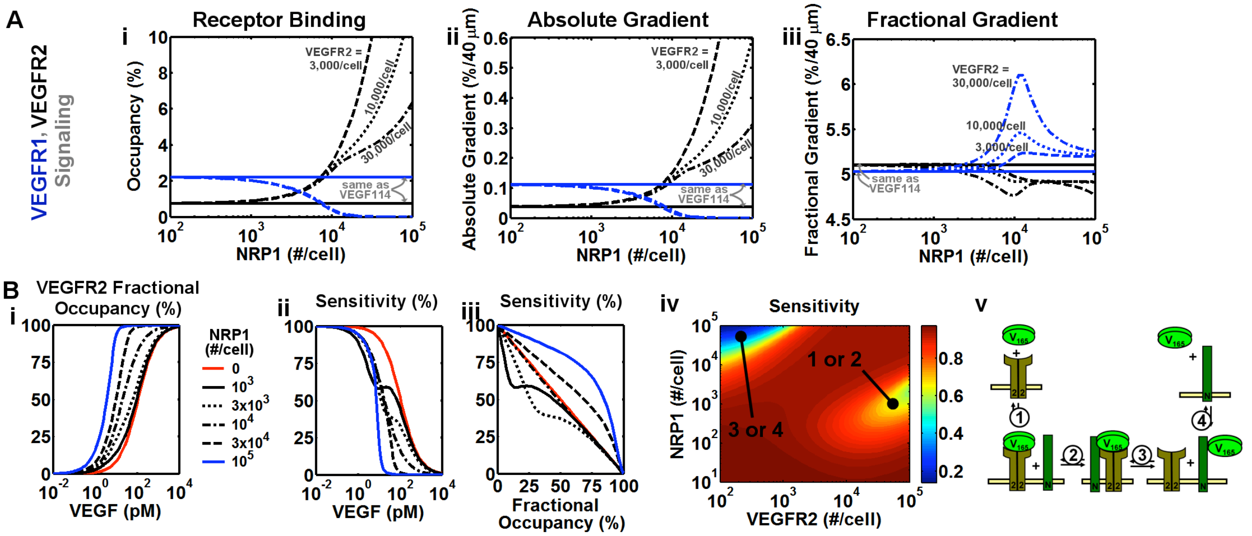

Figure S2.1. Interaction of VEGFR2, VEGFR1, and Neuropilin-1 and mechanism of Neuropilin-1-mediated insensitivity.

A, imposing a VEGF distribution at mean V_0 = 1 pM, g_0 = 5%/40 μm at z = 0 (actual distribution at tip cell, [VEGF] = 0.976 pM, AG: 0.05 pM/40 μm, FG: 5.12%/40 μm), we calculated the signaling response for VEGFR2 (black lines) and VEGFR1 (blue lines) for a range of VEGFR2 ($3{\cdot}10^3$, 10^4, or $3{\cdot}10^4$/cell) and NRP1 (10^2 – 10^5/cell). Responses are given for $VEGF_{165}$, however, solid lines (0 NRP1) coincide with signaling expected for $VEGF_{114}$, which is independent of NRP1. Receptor fractional occupancy (i), absolute gradient of receptor-bound VEGF (ii), and fractional gradient of receptor-bound VEGF (iii). **Aiii**, NRP1 results in a decrease in the VEGFR2-detected fractional gradient (i.e. decreased sensitivity, S_{IFG}) for $VEGF_{165}$.

B, to understand the loss in $VEGF_{165}$ sensitivity to VEGFR2, we measure the VEGF response curve of $VEGF_{165}$-VEGFR2 binding for varying NRP1 (and 10^4 VEGFR2/cell, zero VEGFR1/cell) (i), and calculate sensitivity (ii, iii) using the formula, d log(FO)/d log([VEGF]) (see *Appendix A4*). **Biii**, red line (no NRP1 case) indicates theoretical relation, S = 1 – FO, derived for a one receptor system (see *Section S2.2*). **Biv**, we similarly calculated sensitivity over a range of VEGFR2 and NRP1 expression, at 1 pM $VEGF_{165}$. Contour map indicates two distinct regions of reduced sensitivity. Removing the reactions from the VEGFR2/NRP1 pathway labeled in (v), i.e. both the forward and reverse rates set to zero, led to disappearance of the corresponding local minima in Biv. For all cases, the VEGF concentration was directly imposed and receptors equilibrated, instead of calculating diffusion, to prevent VEGF depletion artifacts near the sprout.

S2.2 Sensitivity of detecting a single VEGF isoform with VEGFR2

Given a particular VEGF distribution, it can be useful to estimate the distribution of receptor activation. We can then estimate the sensitivity of a receptor system to a single VEGF isoform for a given VEGF distribution. Here we neglect VEGF internalization, which allows us to ignore the depletion of VEGF near the cell. In this analysis, we use the differential formulation of the gradient (see Appendix A3). The starting point of the derivation is the equilibrium expression for VEGF binding to VEGFR2:

$$K_d = \frac{\left([R2]_{Total} - [V_{165}R2]\right)[V_{165}]}{[V_{165}R2]} \quad (1)$$

Rearranging gives,

$$[V_{165}R2] = [R2]_{Total}[V_{165}]/([V_{165}] + K_d]) \quad (2)$$

Taking the gradient, we obtain

$$\vec{\nabla}[V_{165}R2] = \frac{[R2]_{Total}K_d}{(K_d + [V_{165}])^2}\vec{\nabla}[V_{165}] \quad (3)$$

And rearranging gives us,

$$\left(\frac{1}{[V_{165}R2]}\vec{\nabla}[V_{165}R2]\right) = \left(\frac{K_d}{K_d + [V_{165}]}\right)\left(\frac{1}{[V_{165}]}\vec{\nabla}[V_{165}]\right) \quad (4)$$

The term $K_d/(K_d+[V_{165}])$ is identified as the sensitivity, $S_{IFG\text{-}165}^{R2}$ the multiplicative factor between the isoform fractional gradients of $VEGF_{165}$ and $VEGF_{165}$-VEGFR2. Note that in this case, the sensitivity is related to the fractional occupancy (FO = $[V_{165}]/(K_d^{V,R2} + [V_{165}])$) by, $S_{IFG\text{-}165} = 1 - FO_{165\text{-}R2}$ and cannot exceed 1.

S2.3 Sensitivity in detecting two VEGF isoforms with a single receptor is dependent on the VEGF gradient

For two VEGF isoforms, the derivation follows similarly. We assume that $VEGF_{165}$ and $VEGF_{114}$ are defined with an arbitrary distribution and that the binding of $VEGF_{165}$ and $VEGF_{114}$ to VEGFR2 is of equal affinity. We follow the derivation for $VEGF_{165}$:

$$K_d = \frac{\left([R2]_{Total} - [V_{165}R2] - [V_{114}R2]\right)[V_{165}]}{[V_{165}R2]} \quad (5)$$

Since this expression holds for $VEGF_{114}$ as well, with $VEGF_{165}$ and $VEGF_{114}$ interchanged, and assuming the affinities of $VEGF_{114}$ and $VEGF_{165}$ to VEGFR2 are identical, we obtain

$$\frac{[V_{165}]}{[V_{165}R2]} = \frac{[V_{114}]}{[V_{114}R2]} \quad (6)$$

This allows us to solve for the concentrations of VEGF-VEGFR2 complexes:

$$[V_{165}R2] = \frac{[V_{165}][R2]_{Total}}{K_d + [V_{165}] + [V_{114}]} \quad (7)$$

$$[V_{114}R2] = \frac{[V_{114}][R2]^{Total}}{K_d + [V_{165}] + [V_{114}]} \quad (8)$$

Taking the gradient of [$VEGF_{165}R2$] gives,

$$\vec{\nabla}[V_{165}R2] = \frac{[V_{165}][R2]_{Total}\left(\vec{\nabla}[V_{165}] + \vec{\nabla}[V_{114}]\right) - [V_{165}][R2]_{Total}\vec{\nabla}[V_{114}]}{\left(K_d + [V_{165}] + [V_{114}]\right)^2} \quad (9)$$

Dividing by [$V_{165}R2$] yields,

$$\frac{1}{[V_{165}R2]}\vec{\nabla}[V_{165}R2] = \frac{K_d + [V_{114}]}{K_d + [V_{165}] + [V_{114}]}\frac{1}{[V_{165}]}\vec{\nabla}[V_{165}] - \frac{1}{K_d + [V_{165}] + [V_{114}]}\vec{\nabla}[V_{114}] \quad (10)$$

or,

$$IFG_{165\text{-}R2} = \frac{K_d + [V_{114}]}{K_d + [V_{165}] + [V_{114}]} IFG_{165} - \frac{[V_{114}]}{K_d + [V_{165}] + [V_{114}]} IFG_{114} \quad (11)$$

The expression for $VEGF_{114}$ is then,

$$IFG_{114\text{-}R2} = \frac{K_d + [V_{165}]}{K_d + [V_{165}] + [V_{114}]} IFG_{114} - \frac{[V_{165}]}{K_d + [V_{165}] + [V_{114}]} IFG_{165} \quad (12)$$

We note that there is no simple expression for the sensitivity of each VEGF isoform in terms of only their VEGF concentrations; surprisingly, the sensitivity of VEGFR2 to any isoform depends on the gradients of all of the isoforms. The sensitivities are determined to be:

$$S^{R2}_{IFG\text{-}165} = \frac{K_d + [V_{114}]\left(1 - IFG_{114}/IFG_{165}\right)}{K_d + [V_{165}] + [V_{114}]} \quad (13)$$

$$S^{R2}_{IFG\text{-}114} = \frac{K_d + [V_{165}]\left(1 - IFG_{165}/IFG_{114}\right)}{K_d + [V_{165}] + [V_{114}]} \quad (14)$$

Note that unless IFG_{165} and IFG_{114} are of opposite signs, it is not possible for sensitivities to exceed 1. The overall sensitivity in the case of one receptor is similar to the sensitivity in the 'one isoform' case by taking the VEGF and VEGF-VEGFR2 concentrations as $VEGF_{165}$ + $VEGF_{114}$ and $VEGF_{165}$-VEGFR2 + $VEGF_{114}$-VEGFR2, respectively, yielding:

$$S^{R2}_{IFG\text{-}VEGF} = \frac{K_d}{K_d + [V_{165}] + [V_{114}]} \quad (15)$$

S2.4 Relationship between individual isoform sensitivities and overall gradients

We can define an overall sensitivity for total soluble VEGF. This is calculated in terms of fractional gradients (and not by IFG) of the individual isoforms, due to the additive nature of FG:

$$S_{IFG\_VEGF} = \frac{IFG_{VEGF\text{-}VEGFR2}}{IFG_{VEGF}} = \frac{FG_{165\text{-}R2} + FG_{121\text{-}R2}}{FG_{165} + FG_{121}} \quad (16)$$

Formulation of sensitivity allows calculation of fractional gradients of VEGF bound to VEGFR2 in terms of isoform fractional gradients of VEGF itself. This is done by noting that

$$AG_{165\text{-}R2} = IFG_{165} \cdot S^{R2}_{IFG\text{-}165} \cdot [V_{165}R2] \quad (17)$$

As a result, the overall fractional gradient of VEGF to VEGFR2 can be expressed as:

$$FG_{VEGF\text{-}R2} = \frac{\left(IFG_{165} \cdot S^{R2}_{IFG\text{-}165} \cdot [V_{165}R2] + IFG_{114} \cdot S^{R2}_{IFG\text{-}114} \cdot [V_{114}R2]\right)}{[V_{165}R2] + [V_{114}R2]} \quad (18)$$

This expression indicates that the overall fractional gradient of VEGF-VEGFR2 is a weighted average of individual VEGF gradients multiplied by the individual sensitivities.

S2.5 Two mechanisms reduce the VEGFR2 sensitivity in detecting $VEGF_{165}$ fractional gradients in the presence of Neuropilin-1

In *Section S2.1*, we showed that formation of the VEGFR2-$VEGF_{165}$-NRP1 ternary complex results in reduced sensitivity of VEGFR2 in detecting $VEGF_{165}$ gradients. In this section, we uncover two specific mechanisms in the balance of VEGFR2 and NRP1 saturation that leads to this predicted behavior.

Since VEGFR2 signaling is mediated by both (or the sum of) $VEGF_{165}$-VEGFR2 and VEGFR2-$VEGF_{165}$-NRP1, we look at the contribution of both binding states to the overall sensitivity:

$$S^{Total\,R2}_{IFG\text{-}165} = \frac{\vec{\nabla}\left([V_{165}R2] + [R2V_{165}N1]\right)}{\vec{\nabla}\left([V_{165}]\right)} \cdot \frac{[V_{165}]}{[V_{165}R2] + [R2V_{165}N1]} \quad (19)$$

Note that this equation can be manipulated to yield

$$S^{Total\,R2}_{IFG\text{-}165} = S^{R2\,alone}_{IFG\text{-}165} \cdot \frac{[V_{165}R2]}{[V_{165}R2] + [R2V_{165}N1]} + S^{R2N1\,alone}_{IFG\text{-}165} \frac{[R2V_{165}N1]}{[V_{165}R2] + [R2V_{165}N1]} \quad (20)$$

Thus, the overall sensitivity is a weighted combination of the sensitivities of the individual VEGFR2 binding states. Since we know the sensitivity of a single isoform to VEGFR2 ($S = K_d/(K_d+[V_{165}])$), it remains to be determined what the sensitivity of the ternary complex to VEGF is. Note that

$$S^{R2N1\,alone}_{IFG-165} = \frac{\vec{\nabla}[R2V_{165}N1]}{\vec{\nabla}[V_{165}]} \cdot \frac{[V_{165}]}{[R2V_{165}N1]} = \left(\frac{\vec{\nabla}[R2V_{165}N1]}{\vec{\nabla}[V_{165}R2]} \cdot \frac{[V_{165}R2]}{[R2V_{165}N1]}\right) \cdot \left(\frac{\vec{\nabla}[V_{165}R2]}{\vec{\nabla}[V_{165}]} \cdot \frac{[V_{165}]}{[V_{165}R2]}\right) \quad (21)$$

or

$$S^{R2N1\,alone}_{IFG-165} = \left(S^{N1}_{IFG-V165R2}\right) \cdot \left(S^{R2\,alone}_{IFG-165}\right) \quad (22)$$

Thus, the overall sensitivity of the ternary complex to $VEGF_{165}$ is simply the sensitivity of $VEGF_{165}$ binding to VEGFR2 coupled to the sensitivity of the $VEGF_{165}$-VEGFR2 complex binding to NRP1. Similar to the case of VEGF binding to a single receptor, to calculate $S_{IFG\text{-}V165R2}^{N1}$, we can assume that NRP1 is a receptor for $VEGF_{165}$-VEGFR2 complex. Since at equilibrium,

$$[R2V_{165}N1] \approx \frac{[N1]_{Total} \cdot [V_{165}R2]}{K^{VR2,N1}_{eq} + [V_{165}R2]} \quad (23)$$

we have that

$S_{IFG\text{-}V165R2}^{N1} = K_{eq}^{VR2,N1}/(K_{eq}^{VR2,N1} + [V_{165}R2])$ (24).

We can now use knowledge of VEGFR2 binding and NRP1 binding to recapitulate the observed decrease in total VEGFR2 sensitivity to $VEGF_{165}$ (Fig. S2.2). The overall sensitivity to all forms of VEGFR2 to $VEGF_{165}$ can be written as

$$S^{\text{Total R2}}_{\text{IFG-165}} = S^{\text{R2 alone}}_{\text{IFG-165}} \cdot \frac{[V_{165}R2]}{[V_{165}R2]+[R2V_{165}N1]} + S^{\text{R2 alone}}_{\text{IFG-165}} \cdot S^{\text{N1}}_{\text{IFG-V165R2}} \cdot \frac{[R2V_{165}N1]}{[V_{165}R2]+[R2V_{165}N1]} \qquad (25)$$

Fig. S2.2Aii shows the profile of $S_{\text{IFG-165}}^{\text{R2 alone}}$ while Fig. S2.2Bii shows $S_{\text{IFG-V165R2}}^{\text{N1}}$. The estimated overall VEGFR2 sensitivity is shown in Fig. S2.2C and agrees well with that observed in Fig. S2.1Biv. For the local minimum at low VEGFR2 and high NRP1, note that VEGFR2 is largely saturated due to formation of the ternary complex and thus $S_{\text{IFG-165}}^{\text{R2 alone}}$ is significantly reduced. At this regime, NRP1 is largely unsaturated. For the local minimum at high VEGFR2 and intermediate NRP1, NRP1 is largely saturated and thus $S_{\text{IFG-V165R2}}^{\text{N1}}$ is small. At low NRP1 levels, [$VEGF_{165}$-VEGFR2] will necessarily be larger than that of the ternary complex, and despite a low NRP1 sensitivity, the overall sensitivity loss is not significant. However, as NRP1 levels increase (to intermediate levels), the ternary complex forms a significant portion of total VEGF-bound VEGFR2 and the loss of overall sensitivity is detectable. Further increases in NRP1 increase $S_{\text{IFG-V165R2}}^{\text{N1}}$ due to NRP1 moving away from saturation.

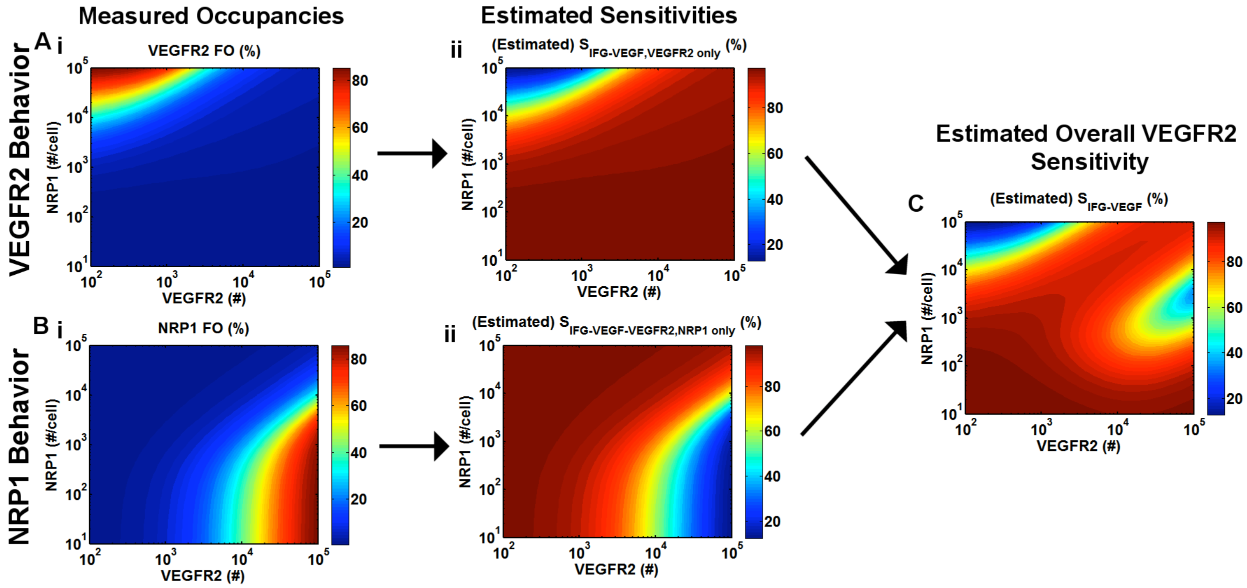

Fig S2.2. VEGFR2 saturation and NRP1 saturation mediate the dual losses in VEGFR2 sensitivity to $VEGF_{165}$ gradients.

We decompose the sensitivity of VEGFR2 as a function of NRP1 and VEGFR2 at $[V_{165}]$ = 1 pM (see Fig. S2.1Biv). **A**, VEGFR2 fractional occupancy (i) and estimated sensitivity to $VEGF_{165}$ (ii) using estimates found in section S2.2. **B**, NRP1 occupancy to $VEGF_{165}$-VEGFR2 (i) allows us to estimate the sensitivity of NRP1 for the $VEGF_{165}$-VEGFR2 complex (ii). **C**, combining the sensitivities as shown in section S2.5 allows us to approximately recapitulate the numerically observed sensitivity profile. The local minima in C are slightly lower than those found in Fig. S2.1Biv, likely due to estimation errors.

Section S3 – Effect of clearance, degradation, and proteolysis on interstitial VEGF levels – compartmental analysis

Our computational model includes several transport processes including HSPG binding, degradation, proteolysis, internalization by the sprout receptors and clearance at the z = -L edge. While our primary model is spatially resolved, we can approximate many of the important results using a simplified compartment model of VEGF transport in the interstitium.

The VEGF in our system exists in three fractions: soluble uncleaved VEGF, V_S; matrix-bound VEGF, V_SH; and cleaved VEGF, V_C. Upon secretion of the uncleaved VEGF at a rate q, VEGF can then bind to HSPGs, at rate constants $k_{on}^{V,H}$ and $k_{off}^{V,H}$, be degraded at a rate k_{deg}, cleared at a rate k_C, or proteolyzed at a rate k_P:

$$d[V_S]/dt = q - k_{on}^{V,H}[V_S][H] + k_{off}^{V,H}[V_SH] - k_{deg}^{S}[V_S] - k_C^S[V_S] - k_P^S[V_S] \quad (1)$$

$$d[V_SH]/dt = k_{on}^{V,H}[V_S][H] - k_{off}^{V,H}[V_SH] - k_{deg}^{H}[V_SH] - k_P^H[V_SH] \quad (2)$$

$$d[V_C]/dt = -k_{deg}^{C}[V_C] - k_C^C[V_C] + k_P^S[V_S] + k_P^H[V_SH] \quad (3)$$

In these equations, subscripts denote the reaction involved (deg for degradation, P for proteolysis, C for clearance) while superscripts denote which species participate in the reaction (S for soluble uncleaved VEGF, C for cleaved VEGF, and H for matrix-bound VEGF). We can solve the steady-state solution of this general system by assuming that soluble VEGF levels are low (i.e. $[V_S] << K_d^{V,H}$ or that $[H] \sim [H]_{Total}$) to get:

$$[V_S] = \frac{q}{k_{deg}^S + k_P^S + k_C^S + \frac{(k_{deg}^H + k_P^H)\cdot k_{on}^{V,H}[H]_{Total}}{k_{off}^{V,H} + k_{deg}^H + k_P^H}} \quad (4)$$

$$[V_SH] = k_{on}^{V,H}[V_S][H]_{Total}/(k_{off}^{V,H} + k_{deg}^H + k_P^H) \quad (5)$$

$$[V_C] = (k_P^S[V_S] + k_P^H[V_SH])/(k_{deg}^C + k_C^C) \quad (6)$$

The steady-state total VEGF is then given by

$$[V_{Total}] = \frac{q}{k_{deg}^S + k_P^S + k_C^S + \frac{(k_{deg}^H + k_P^H)\cdot k_{on}^{V,H}[H]_{Total}}{k_{off}^{V,H} + k_{deg}^H + k_P^H}} \cdot \left[1 + \frac{k_{on}^{V,H}[H]_{Total}}{k_{off}^{V,H} + k_{deg}^H + k_P^H} + \frac{k_P^S}{k_{deg}^C + k_C^C} + \frac{k_{on}^{V,H}[H]_{Total}}{k_{off}^{V,H} + k_{deg}^H + k_P^H}\cdot\frac{k_P^H}{k_{deg}^C + k_C^C}\right] \quad (7)$$

Notice the form of the equation 7: $[V_{Total}]$ = S+SH+SC+SHC, where S is soluble uncleaved VEGF, SH is bound VEGF, SC is the cleaved VEGF originating from S, and SHC is the cleaved VEGF originating from SH. This provides a basis for the proportionality of soluble uncleaved and matrix-bound VEGF fractions seen in the manuscript (Fig. 4). Uncleaved VEGF is decreased not only by processes that affect the uncleaved soluble VEGF directly, but also by processes that affect HSPG-bound VEGF.

We are interested in the limiting behaviors of this system. For example, the HSPG-binding-only model is replicated by assuming k_{deg} = 0, in which case $[V_S]+[V_C] = q/k_C$, i.e. VEGF levels are determined only by the total clearance rate. This result is also consistent with

the lack of influence of HSPGs on the soluble VEGF distribution. Isoform-independent degradation is given by assuming k_{deg}^S is nonzero, while k_{deg}^H is 0, and in this case, $[V_S]+[V_C] = q/(k_C + k_{deg})$. When clearance is not present but isoform-specific degradation is present (k_{deg} for all fractions is nonzero), we have the interesting result that $[V_{Total}] = q/k_{deg}$ (if all fractions have the same degradation rates). Note that this expression indicates that isoform-specific degradation can preserve total VEGF levels in a tissue, independent of varying isoform affinities to HSPGs and proteolysis rates.

Note generally that for any species [X] = (sum of sources)/(sum of sinks) at steady-state. This implies that the steady-state concentration of any species depends on the average time a secreted VEGF molecule spends in that state before it is eventually removed by its sinks. This is an invocation of the ergodic hypothesis: time-averaging the state occupied by an individual VEGF molecule is equivalent to the proportion of VEGF occupied in each state. For example, $[V_{Total}]$ indicates the total amount of time a secreted VEGF molecule spends in the entire system (i.e. the residence time of the entire system), while $[V_S]+[V_C]$ indicates the amount of time a VEGF molecule spends in solution until it is cleared or degraded. In the HSPG-binding-only model, we observe that total VEGF is significantly increased by HSPG binding (i.e. proportional to the HSPG affinity), which indicates that residence time of VEGF is significantly increased by HSPGs. Since the rate of VEGF association to HSPGs is equal to the sum of bound VEGF dissociation and cleavage at steady-state, the amount of time VEGF spends in solution is independent of HSPGs. Similarly, the fact that $[V_{Total}]$ is constant in the isoform-specific degradation model (in the absence of clearance) indicates that VEGF molecules spend the same amount of time in the domain, which follows as no state is free from the effect of degradation. This explains why cleaved VEGF levels are relatively independent of HSPG affinity in the isoform-specific degradation model (see Fig. 6Ci of manuscript), as different VEGF isoforms would spend the same amount of time in the system, time in which they are exposed to the influence of proteases.

Section S4 – Extended results for isoform-specific degradation model

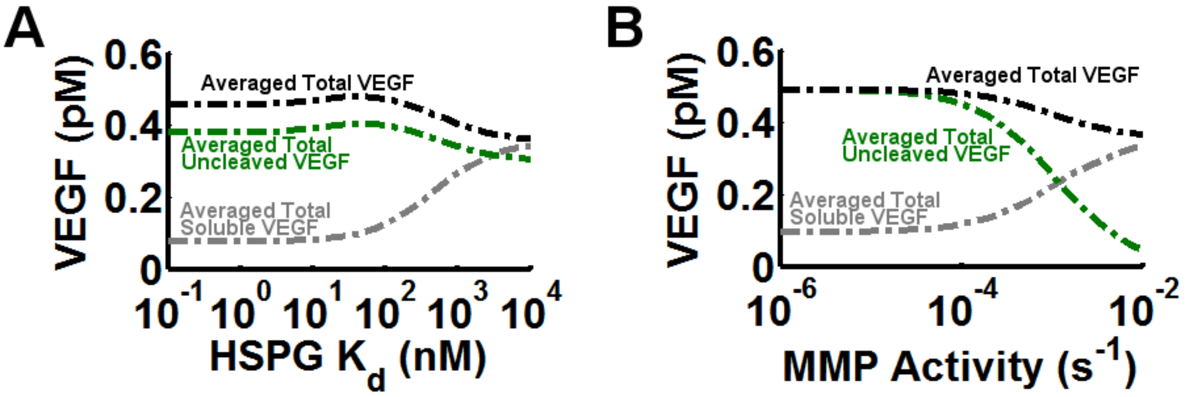

Fig. S4.1. Total VEGF in the domain is well preserved as isoform affinity and protease activity varies.
We plot the averaged VEGF concentration in the domain of total soluble fractions (gray lines), total uncleaved fractions (green lines), and total VEGF (black lines). The extent of mass conservation is indicated by the range of the contour plots. For total soluble VEGF and total uncleaved VEGF, they vary between 0 and their maximal values; however for total VEGF, the minimal to maximal value ranges only ~40%.

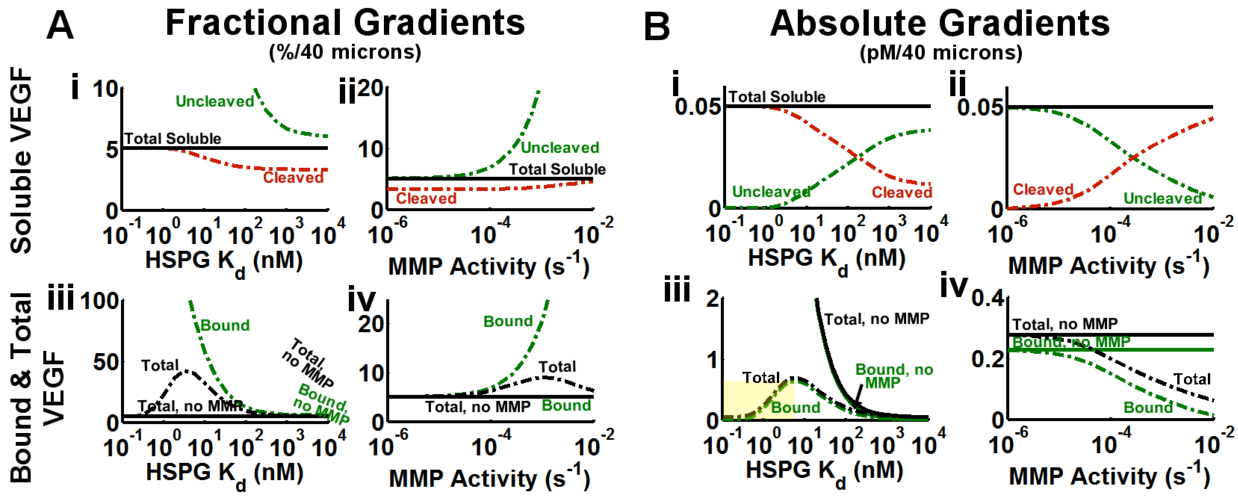

Fig. S4.2. VEGF gradients in the HSPG-binding-only model.
This section was calculated for comparison to the isoform-specific degradation model (Fig. S4.3). To understand if VEGF fractional gradients (**A**) and absolute gradients (**B**) can recapitulate the isoform monotonicity and HSPG/MMP antagonism of vascular patterning, we calculated their dependence to varying the isoform (i,iii) and MMP activity levels (ii,iv). Analysis is similar to Fig. 6 of manuscript. In this model, the absolute gradient of matrix-bound VEGF (Biii) exhibits biphasic behavior similar to the concentration (Fig. 6Biii of manuscript), however the region of monotonicity extends to one decade higher affinities, making it a potentially superior indicator than the concentration itself. Interestingly, the fractional gradient of total VEGF shows two separate regions where HSPG/MMP antagonism is seen, with a large region where the antagonism is broken indicating that this signal is not sufficient to guide vascular patterning. All other signals show negative behaviors.

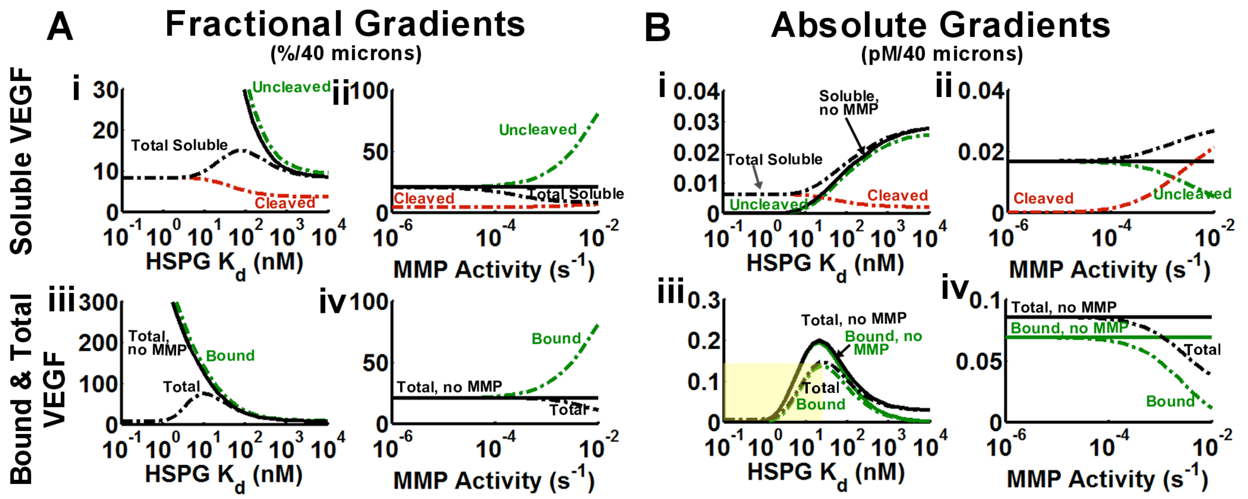

Fig. S4.3. VEGF gradients in the isoform-specific degradation model.

To understand if VEGF fractional gradients (**A**) and absolute gradients (**B**) can recapitulate the isoform monotonicity and HSPG/MMP antagonism of vascular patterning, we calculated their dependence to varying the isoform (i,iii) and MMP activity levels (ii,iv). Analysis is similar to Fig. 6 of manuscript. While the fractional gradient of total VEGF (Aiii,iv) obeys both isoform monotonicity and HSPG/MMP antagonism over a modest range of isoform affinities, note that its dynamic range in the variation of MMP activity (Aiv) is significantly smaller than that found when varying isoforms (Aiii) and hence it is likely of marginal use in recovering vascular patterning. The dynamic range of the absolute gradient of total soluble VEGF in its variation in MMP activity is less restrictive however is not as effective as the total soluble concentration (Fig. 6Ci,ii of manuscript).

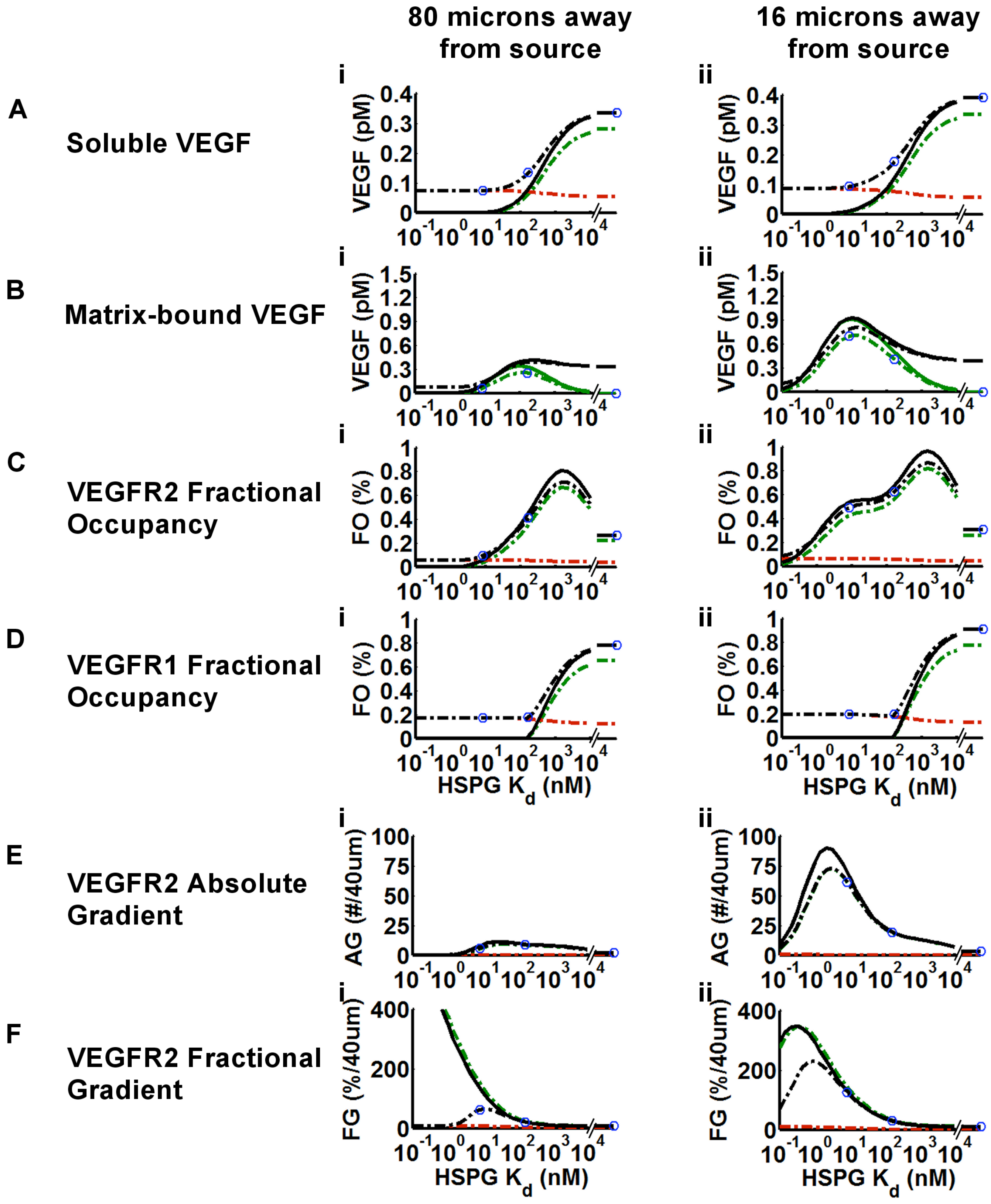

Fig. S4.4. Effect of the sprout location on isoform monotonicity of VEGF signaling modes.
Various VEGF sensing modes (**A-F**) were considered with respect to the HSPG affinity of the isoform that is secreted, at two spatial positions: the tip of the tip cell 80 µm away from the secretion source (i) and 16 µm away from the secretion source (ii). Note that ordering of isoforms through matrix-bound VEGF changes when the sensing occurs close to the secretion source (**B**). While it appears that the ordering of isoforms detected through VEGFR2 signaling and VEGFR2 absolute gradients also changes when the sensing occurs closer to the secretion source, this behavior is dependent upon specific binding parameters on how NRP1 binding affinity was varied with respect to the isoforms (*not shown*). Thus, the effect may not hold in all cases.

References

1. Krilleke D, DeErkenez A, Schubert W, Giri I, Robinson GS, Ng YS, Shima DT: **Molecular mapping and functional characterization of the VEGF164 heparin-binding domain**. *J Biol Chem* 2007, **282**(38):28045-28056.
2. Ashikari-Hada S, Habuchi H, Kariya Y, Itoh N, Reddi AH, Kimata K: **Characterization of growth factor-binding structures in heparin/heparan sulfate using an octasaccharide library**. *J Biol Chem* 2004, **279**(13):12346-12354.
3. Houck KA, Leung DW, Rowland AM, Winer J, Ferrara N: **Dual regulation of vascular endothelial growth factor bioavailability by genetic and proteolytic mechanisms**. *J Biol Chem* 1992, **267**(36):26031-26037.
4. Tomii Y, Yamazaki H, Sawa N, Ohnishi Y, Kamochi J, Tokunaga T, Osamura Y, Sadahiro S, Kijima H, Abe Y *et al*: **Unique properties of 189 amino acid isoform of vascular endothelial growth factor in tumorigenesis**. *Int J Oncol* 2002, **21**(6):1251-1257.
5. Zhang HT, Scott PA, Morbidelli L, Peak S, Moore J, Turley H, Harris AL, Ziche M, Bicknell R: **The 121 amino acid isoform of vascular endothelial growth factor is more strongly tumorigenic than other splice variants in vivo**. *Br J Cancer* 2000, **83**(1):63-68.
6. Dowd CJ, Cooney CL, Nugent MA: **Heparan sulfate mediates bFGF transport through basement membrane by diffusion with rapid reversible binding**. *J Biol Chem* 1999, **274**(8):5236-5244.
7. Cebe-Suarez S, Grunewald FS, Jaussi R, Li X, Claesson-Welsh L, Spillmann D, Mercer AA, Prota AE, Ballmer-Hofer K: **Orf virus VEGF-E NZ2 promotes paracellular NRP-1/VEGFR-2 coreceptor assembly via the peptide RPPR**. *FASEB J* 2008, **22**(8):3078-3086.
8. Templeton DM: **Proteoglycans in cell regulation**. *Crit Rev Clin Lab Sci* 1992, **29**(2):141-184.
9. Press WH, Teukolsky SA, Vetterling WT, Flannery BP: **Numerical Recipes in C++: Art of Scientific Computing**, 2nd edn. New York: Cambridge University Press; 2002.
10. Thompson LD, Pantoliano MW, Springer BA: **Energetic characterization of the basic fibroblast growth factor-heparin interaction: identification of the heparin binding domain**. *Biochemistry* 1994, **33**(13):3831-3840.
11. Keyt BA, Berleau LT, Nguyen HV, Chen H, Heinsohn H, Vandlen R, Ferrara N: **The carboxyl-terminal domain (111-165) of vascular endothelial growth factor is critical for its mitogenic potency**. *J Biol Chem* 1996, **271**(13):7788-7795.
12. Robinson CJ, Mulloy B, Gallagher JT, Stringer SE: **VEGF165-binding sites within heparan sulfate encompass two highly sulfated domains and can be liberated by K5 lyase**. *J Biol Chem* 2006, **281**(3):1731-1740.
13. Plouet J, Moro F, Bertagnolli S, Coldeboeuf N, Mazarguil H, Clamens S, Bayard F: **Extracellular cleavage of the vascular endothelial growth factor 189-amino acid form by urokinase is required for its mitogenic effect**. *J Biol Chem* 1997, **272**(20):13390-13396.
14. Jonca F, Ortega N, Gleizes PE, Bertrand N, Plouet J: **Cell release of bioactive fibroblast growth factor 2 by exon 6-encoded sequence of vascular endothelial growth factor**. *J Biol Chem* 1997, **272**(39):24203-24209.
15. Poltorak Z, Cohen T, Sivan R, Kandelis Y, Spira G, Vlodavsky I, Keshet E, Neufeld G: **VEGF145, a secreted vascular endothelial growth factor isoform that binds to extracellular matrix**. *J Biol Chem* 1997, **272**(11):7151-7158.
16. Kawamura H, Li X, Harper SJ, Bates DO, Claesson-Welsh L: **Vascular endothelial growth factor (VEGF)-A165b is a weak in vitro agonist for VEGF receptor-2 due to lack of coreceptor binding and deficient regulation of kinase activity**. *Cancer Res* 2008, **68**(12):4683-4692.

17. Lange T, Guttmann-Raviv N, Baruch L, Machluf M, Neufeld G: **VEGF162, a new heparin-binding vascular endothelial growth factor splice form that is expressed in transformed human cells**. *J Biol Chem* 2003, **278**(19):17164-17169.
18. Gengrinovitch S, Berman B, David G, Witte L, Neufeld G, Ron D: **Glypican-1 is a VEGF165 binding proteoglycan that acts as an extracellular chaperone for VEGF165**. *J Biol Chem* 1999, **274**(16):10816-10822.
19. Jones M, Tussey L, Athanasou N, Jackson DG: **Heparan sulfate proteoglycan isoforms of the CD44 hyaluronan receptor induced in human inflammatory macrophages can function as paracrine regulators of fibroblast growth factor action**. *J Biol Chem* 2000, **275**(11):7964-7974.
20. Gupta K, Gupta P, Wild R, Ramakrishnan S, Hebbel RP: **Binding and displacement of vascular endothelial growth factor (VEGF) by thrombospondin: effect on human microvascular endothelial cell proliferation and angiogenesis**. *Angiogenesis* 1999, **3**(2):147-158.
21. Lake AC, Vassy R, Di Benedetto M, Lavigne D, Le Visage C, Perret GY, Letourneur D: **Low molecular weight fucoidan increases VEGF165-induced endothelial cell migration by enhancing VEGF165 binding to VEGFR-2 and NRP1**. *J Biol Chem* 2006, **281**(49):37844-37852.
22. Gospodarowicz D, Cheng J, Lui GM, Baird A, Bohlent P: **Isolation of brain fibroblast growth factor by heparin-Sepharose affinity chromatography: identity with pituitary fibroblast growth factor**. *Proc Natl Acad Sci U S A* 1984, **81**(22):6963-6967.
23. Baird A, Ling N: **Fibroblast growth factors are present in the extracellular matrix produced by endothelial cells in vitro: implications for a role of heparinase-like enzymes in the neovascular response**. *Biochem Biophys Res Commun* 1987, **142**(2):428-435.
24. Whitelock JM, Murdoch AD, Iozzo RV, Underwood PA: **The degradation of human endothelial cell-derived perlecan and release of bound basic fibroblast growth factor by stromelysin, collagenase, plasmin, and heparanases**. *J Biol Chem* 1996, **271**(17):10079-10086.
25. Nugent MA, Edelman ER: **Kinetics of basic fibroblast growth factor binding to its receptor and heparan sulfate proteoglycan: a mechanism for cooperactivity**. *Biochemistry* 1992, **31**(37):8876-8883.
26. Whitelock JM, Graham LD, Melrose J, Murdoch AD, Iozzo RV, Underwood PA: **Human perlecan immunopurified from different endothelial cell sources has different adhesive properties for vascular cells**. *Matrix Biol* 1999, **18**(2):163-178.
27. Pan Q, Chathery Y, Wu Y, Rathore N, Tong RK, Peale F, Bagri A, Tessier-Lavigne M, Koch AW, Watts RJ: **Neuropilin-1 binds to VEGF121 and regulates endothelial cell migration and sprouting**. *J Biol Chem* 2007, **282**(33):24049-24056.
28. Fuh G, Garcia KC, de Vos AM: **The interaction of neuropilin-1 with vascular endothelial growth factor and its receptor flt-1**. *J Biol Chem* 2000, **275**(35):26690-26695.
